# Uncoordinated maturation of developing and regenerating postnatal mammalian vestibular hair cells

**DOI:** 10.1371/journal.pbio.3000326

**Published:** 2019-07-01

**Authors:** Tian Wang, Mamiko Niwa, Zahra N. Sayyid, Davood K. Hosseini, Nicole Pham, Sherri M. Jones, Anthony J. Ricci, Alan G. Cheng

**Affiliations:** 1 Department of Otolaryngology-Head and Neck Surgery, Stanford University School of Medicine, Stanford, California, United States of America; 2 Department of Otolaryngology-Head and Neck Surgery, The Second Xiangya Hospital, Central South University, Changsha, Hunan, China; 3 Department of Special Education and Communication Disorders, College of Education and Human Sciences, University of Nebraska, Lincoln, Nebraska, United States of America; 4 Department of Molecular and Cellular Physiology, Stanford University School of Medicine, Stanford, California, United States of America; The University of Sheffield, UNITED KINGDOM

## Abstract

Sensory hair cells are mechanoreceptors required for hearing and balance functions. From embryonic development, hair cells acquire apical stereociliary bundles for mechanosensation, basolateral ion channels that shape receptor potential, and synaptic contacts for conveying information centrally. These key maturation steps are sequential and presumed coupled; however, whether hair cells emerging postnatally mature similarly is unknown. Here, we show that in vivo postnatally generated and regenerated hair cells in the utricle, a vestibular organ detecting linear acceleration, acquired some mature somatic features but hair bundles appeared nonfunctional and short. The utricle consists of two hair cell subtypes with distinct morphological, electrophysiological and synaptic features. In both the undamaged and damaged utricle, fate-mapping and electrophysiology experiments showed that Plp1^+^ supporting cells took on type II hair cell properties based on molecular markers, basolateral conductances and synaptic properties yet stereociliary bundles were absent, or small and nonfunctional. By contrast, Lgr5^+^ supporting cells regenerated hair cells with type I and II properties, representing a distinct hair cell precursor subtype. Lastly, direct physiological measurements showed that utricular function abolished by damage was partially regained during regeneration. Together, our data reveal a previously unrecognized aberrant maturation program for hair cells generated and regenerated postnatally and may have broad implications for inner ear regenerative therapies.

## Abstract 2

During development, sensory hair cells undergo a series of critical maturation steps that are sequential and presumed coupled, but whether regenerated hair cells mature similarly is unknown. This study shows that regenerated vestibular hair cells acquired some mature somatic features, but the apical bundles remained immature.

## Introduction

Inner ear hair cells are mechanoreceptors critical for auditory and vestibular functions, and their degeneration are primary causes of hearing and balance dysfunction. In mammals, cochlear hair cell loss is irreversible, leading to permanent hearing loss [1]. By contrast, spontaneous yet limited hair cell regeneration occurs in both the neonatal and mature mammalian utricle [2–10], a vestibular sensory organ that functions to detect linear acceleration such as gravity.

During utricular development, hair cells are specified between embryonic day (E) 11.5–12.5 [11]. At birth, some hair cells exhibit mature-appearing stereociliary bundles bearing the actin-bundling protein espin [12]. Electrophysiological measurements show that vestibular hair cells are mechanically sensitive as early as E16 [13]. Outward and inward rectifier voltage-dependent conductances characteristic of type I and II hair cells are present before birth, and their properties continue to mature during the first two postnatal weeks [14–16]. Formation of a calyx, a specialized nerve terminal specific for type I hair cells, begins neonatally and is presumed to be mature within the first postnatal month [16]. Thus, within four weeks of specification, utricular hair cells can first display mature bundle and then somatic (electrophysiological, synaptic, and molecular) features representing specialization of hair cell subtypes. These properties, which are presumed to be orderly and coupled, are used as references for characterizing induced pluripotent stem (iPS) cell- and progenitor cell-derived hair cells [17–20]. However, over 50% of utricular hair cells are added after birth, with most addition occurring during the first postnatal week when the organ gains detectable function [21,22]. At present, the time course and extent of functional maturation acquired by postnatally developed hair cells are unexplored, leaving open the question of whether they display properties similar to those born in the embryonic period.

Following damage to the mammalian utricle, hair cells bearing immature stereociliary bundles reminiscent of nascent hair cells repopulate the sensory epithelium one to six months later [3–6,8–10]. In the mature mouse utricle, the extent of regenerated hair cells is rather limited, whereas more robust regeneration occurs in utricles from younger animals [5,9]. Using length of stereociliary bundles as a surrogate marker of maturation, most regenerated hair cells appear nascent even months after damage [5]. Given this limited degree of hair cell regeneration and bundle maturation, it is currently unclear whether the somatic properties (basolateral conductances, synaptic/nerve terminal formation and molecular markers) of regenerated hair cells mature and if the overall vestibular function is restored.

As in other vestibular organs, hair cells in the utricle specialize into type I and II subtypes, which can be distinguished by molecular markers (Osteopontin for mature type I, Mapt for mature type II and Annexin A4 for immature and mature type II) [23], morphology of cell body (amphora-shaped with apical neck for type I and cylindrical or goblet-shaped for type II), nerve terminals (calyces for type I and boutons for type II), and basolateral conductances (low-voltage-activated, delayed rectifier potassium conductance [gKL] for type I and slow inward rectifier non-specific cation conductance [gH] for both type I and II) [24,14,25–29]. Type I hair cells display larger mechanotransducer currents and are postulated to detect faster acceleration stimuli [30–32], but both subtypes are deemed essential for organ function [33]. In the damaged mature mammalian utricle, regeneration of only type II hair cells has been reported [3–6], whereas hair cells regenerated in the neonatal utricle displayed molecular characteristics of both type I and II hair cells [9]. At present, the extent of maturity achieved by postnatally generated and regenerated hair cells into hair cell subtypes—including their acquisition of basolateral conductances, nerve terminals, and molecular markers—has not been fully defined in either the neonatal or mature utricle.

Here, we found that postnatally generated hair cells displayed electrophysiological and molecular characteristics of type II hair cells but had relatively immature stereociliary bundles. Using lineage markers differentially expressed in extrastriolar (peripheral) and striolar (central) supporting cells [9], we show that regenerated hair cells derived from Plp1^+^ supporting cells displayed type II hair cell properties whereas those from the Lgr5^+^ lineage exhibited either type I or II hair cell properties, suggesting regional segregation of hair cell precursors. In contrast to somatic properties (morphology, hair cell subtype markers, synaptic markers, nerve terminals, and basolateral conductances), regenerated hair cells showed relatively immature or nonfunctional bundle characteristics (morphology and mechanotransduction [MET] channel function). Collectively, our study reveals that postnatally generated and regenerated hair cells acquire many somatic properties but retain relatively immature bundle features, thus displaying previously unrecognized aberrant maturation programs distinct from embryonic hair cells.

## Results

### Fate-mapping early and late postnatally generated hair cells

**Fig 1:**
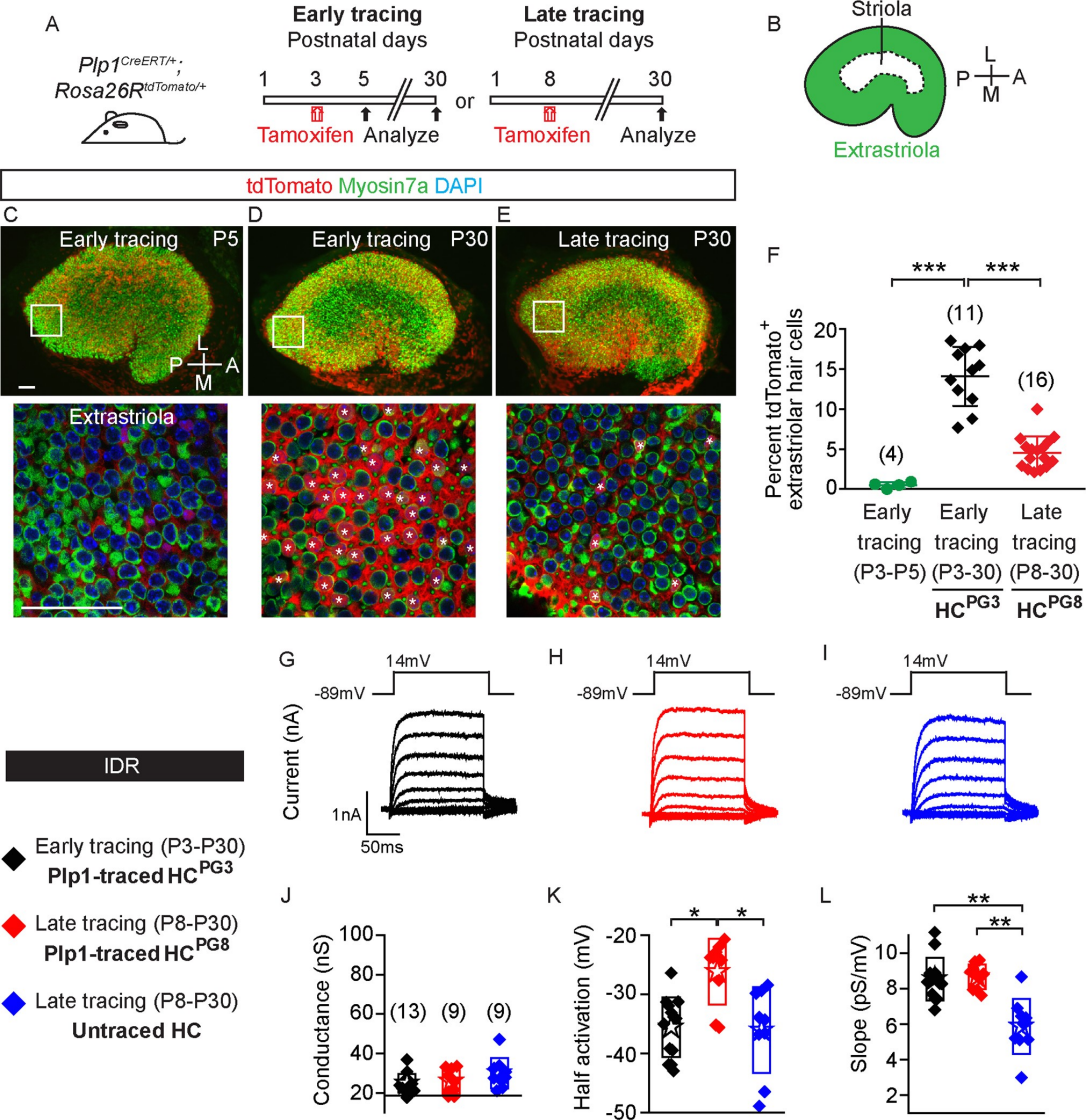
Characteristics of postnatally generated hair cells in the mouse utricle. A) *Plp1*^*CreERT/+*^*; Rosa26R*^*tdTomato/+*^ mice were treated with tamoxifen at P3 (early tracing) and P8 (late tracing) to fate-map supporting cells. Organs were examined at P5 and P30. B) Diagram illustrating the extrastriolar and striolar regions of the utricle. C) Rare tdTomato^+^/Myosin7a^+^ hair cells were detected in the extrastriola 2 days after early tracing with tamoxifen given at P3. D) Many traced hair cells (asterisks) were found in the extrastriola at P30. E) After late tracing initiated at P8, few traced hair cells (asterisks) were detected in the extrastriola at P30. Representative high magnification images were selected from the extrastriola region (white boxes). F) Compared to P5, the percentage of tdTomato^+^/Myosin7a^+^ cells was significantly higher at P30 after early tracing from P3. It is also significantly higher than that of late tracing from P8 (*n =* 4–16 mice). G-I) Currents from hair cells were elicited using the delayed rectifier protocol described in the methods. J-L) Conductance-voltage plots were generated and values for maximal conductance, half-activation voltage and slope of the Boltzmann function were extracted (*n =* 9–13 cells). All examined hair cells from three groups displayed measurable conductances representative of gDR, with some significant differences in half-activation voltage and slope. Data shown as mean ± SD, compared using Student *t* tests and one-way ANOVA by Kruskal Wallis-Dunn's multiple comparison tests. ***p < 0.001, **p < 0.01, *p < 0.05. Scale bars: 50 μm. The underlying data can be found within S1 Data. P, postnatal day.

In the postnatal mouse utricle, most hair cells are added during the first week after birth in both the striolar and extrastriolar regions [21]. To label the postnatally generated hair cells (HC^PG^), we fate-mapped supporting cells in *Plp1*^*CreERT/+*^*; Rosa26R*^*tdTomato/+*^ (*Plp1-Tomato*) mice (S1A Fig) [9]. Tamoxifen (0.075 mg/g intraperitoneal [IP]) administration at postnatal day (P) 3 induced tdTomato labeling of 73.0 ± 10.3% and 14.1 ± 4.0% supporting cells in the extrastriolar and striolar regions, respectively, two days later (*n =* 4, Fig 1A–1C, S1A and S1B Fig). Hair cells were rarely tdTomato-labeled at this age (0.5 ± 0.3 and 0.3 ± 0.5%, *n =* 742 extrastriolar and 663 striolar hair cells from 4 mice) (Fig 1C and 1F, S1B and S1E Fig). At P30, 14.1 ± 3.7% of Myosin7a^+^ hair cells in the extrastriola and 5.2 ± 3.7% in the striola were tdTomato-labeled (*n =* 2,507 extrastriolar and 1,858 striolar hair cells from 11 mice, Fig 1D and 1F, S1C and S1E Fig), both significantly more than P5 (*p* < 0.001 in extrastriola and *p* < 0.01 in striola). Control experiments using corn oil on P3 *Plp1-tdTomato* mice revealed no labeled hair cells (*n =* 665 and 608 hair cells in the extrastriola and striola from 3 mice) and only rare labeled supporting cells at P30 (0.5% of 954 extrastriolar and 0.1% of 946 striolar supporting cells from 3 mice, S1F Fig). This suggests that P3 Plp1^+^ supporting cells contribute to postnatally generated hair cells, which we term HC^PG3^ hereon.

The number of hair cells in the mouse utricle continues to increase during the first postnatal week, with marginal increases thereafter [21]. To identify these late postnatally generated hair cells, we administered tamoxifen (0.075 mg/g IP) to P8 *Plp1-Tomato* mice and found 4.6 ± 2.0% and 2.5 ± 2.5% of Myosin7a^+^ hair cells tdTomato-labeled in the extrastriola and striola, respectively, at P30 (*n =* 3,002 extrastriolar and 2,561 striolar hair cells from 16 mice, Fig 1E and 1F, S1D and S1E Fig). There were significantly fewer late traced hair cells (HC^PG8^) than HC^PG3^ (Fig 1F, S1E Fig, S1 Data), consistent with the decline in hair cell production after the first postnatal week [21]. To assess the electrophysiological properties of HC^PG3^ and HC^PG8^, we probed both cohorts at P30, allowing time for both groups of hair cells to mature. Traced hair cells (HC^PG3^ and HC^PG8^) and untraced hair cells (controls), which likely contained more mature hair cells derived from supporting cells earlier than P8, were probed for delayed rectifier potassium currents (IDR). Normally found in nascent and mature vestibular hair cells [14,16], IDR were observed in all three groups of hair cells (*n =* 13 HC^PG3^, 9 HC^PG8^, 9 untraced hair cells, Fig 1G–1I). From conductance-voltage plots, we found that values for maximal conductance, half-activation voltage and slope of the Boltzmann function were similar among groups (Fig 1J–1L, S1 Data), with small but significant differences among the latter two measurements possibly attributed to splice variants or phosphorylation state. Lastly, we used two additional measurements to estimate hair cell maturation: 1) cell capacitance as a proxy for cell size and 2) resting potentials which become more negative as hair cells mature [15]. We found that traced hair cells (HC^PG3^ and HC^PG8^) most closely matched perinatal hair cells in both size and resting potentials and estimate that they were comparably mature (S2 Table) [15]. Together, these data show that both early and late postnatally generated hair cells exhibit fundamental hair cell properties, leading us next to probe whether they acquire specialized hair cell subtype characteristics.

### Specialization of postnatally generated hair cells into type II hair cells

Vestibular hair cell subtypes can be identified based on morphology, molecular markers, electrophysiological and synaptic properties. In addition, hair cell age can be intimated by the complement of ion channels present, synaptic function, and the morphology and functional state of hair bundles. Here, we first used molecular markers and electrophysiological properties to determine whether traced hair cells differentiate into hair cell subtypes.

Type I hair cells display amphora-shaped cell bodies and long thin apical necks, endowed with a consistent and simple electrophysiological fingerprint: a hyperpolarized activating delayed rectifier current (IKL) [16]. Type II hair cells are goblet-shaped with short, thick necks and basolateral cytoplasmic processes, but are more complex in that they contain diverse ion channels that vary with age and location, including the voltage-gated inward rectifying, nonspecific cation currents (IH) [26,16].

**Fig 2:**
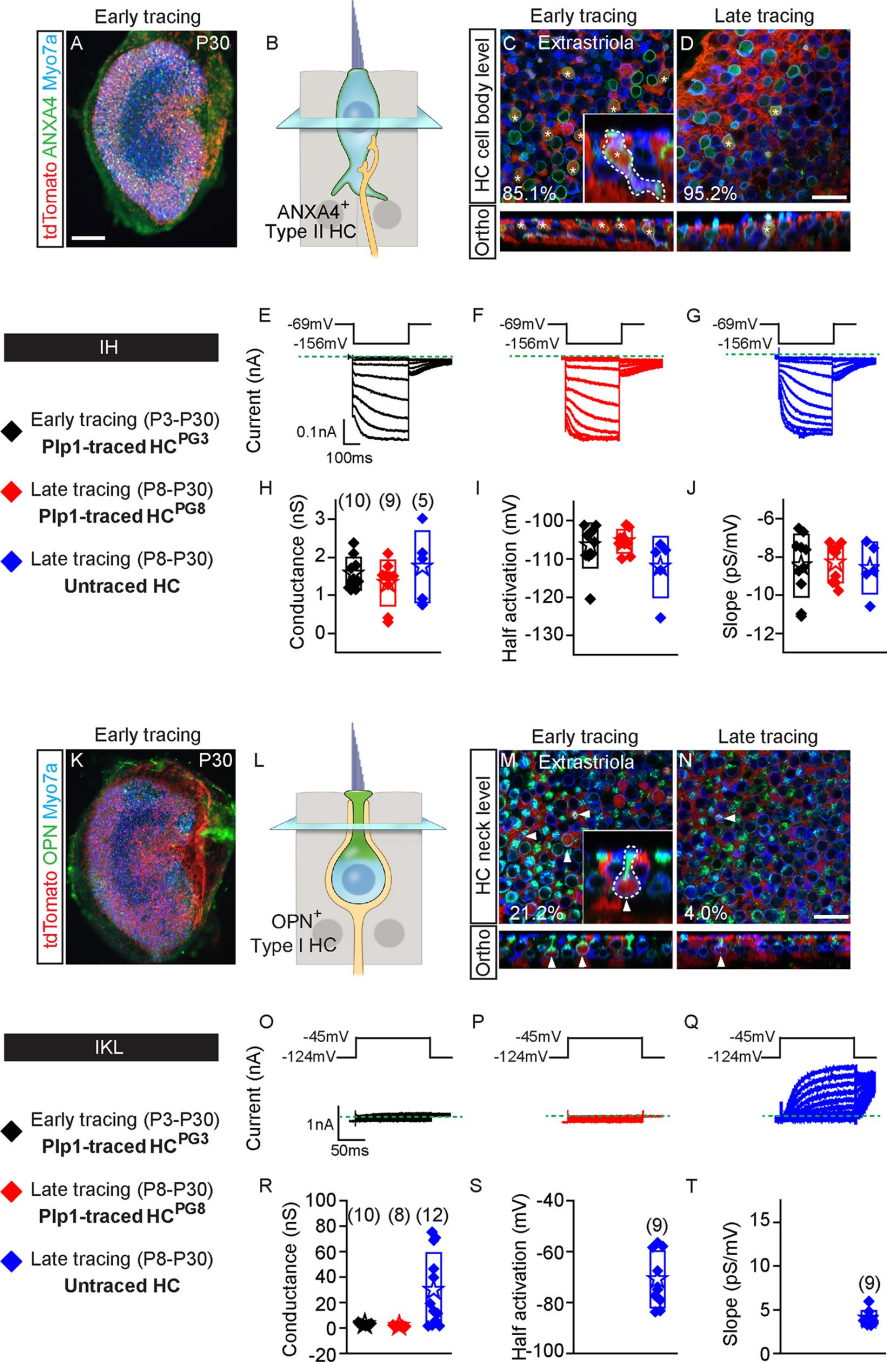
Early and late postnatally generated hair cells primarily show type II hair cell characteristics. A) P30 whole mount utricle (early tracing) labeled for Annexin A4 (ANXA4). B) Cartoon depicting nucleus level of ANXA4^+^ (green) type II hair cells. C-D) Almost all postnatally generated tdTomato^+^/Myosin7a^+^ hair cells from early and late tracing expressed ANXA4 (asterisks). Inset shows orthogonal view of traced goblet-shaped, ANXA4^+^ type II hair cells with basolateral processes. E-G) Representative tracings of voltage-gated currents in response to the inward rectifier protocol from HC^PG3^, HC^PG8^ and untraced hair cells (*n =* 5–10 cells). H-J) All three groups demonstrate similar inward rectifier electrophysiological (gH) properties: peak conductances, half-activation and slopes. K) P30 utricle from early tracing labeled for OPN. L) Diagram illustrating the nucleus level of OPN^+^ (green) type I hair cells. Occasional tdTomato^+^/Myosin7a^+^ hair cells from early M) and late tracing N) expressed OPN on the apical neck (arrowhead). Inset shows orthogonal view of traced OPN^+^ type I hair cells with amphora shape, and a long and narrow apical neck. O-Q) Examples of low voltage activated potassium current (IKL) responses during the step displacements for HC^PG3^, HC^PG8^ and untraced hair cells (*n =* 8–12 cells). Green dashed lines define zero current levels. R-T) Similar tail current and reversal potential analysis were performed with resultant data for conductance, half-activation and slope. Data shown as mean ± SD, compared using one-way ANOVA by Kruskal Wallis-Dunn's multiple comparison test. Scale bars: A, K) 100 μm; C-D, M-N) 20 μm. The underlying data can be found within S1 Data. ANXA4, Annexin A4; HC^PG^, postnatally generated hair cell; OPN, Osteopontin; P, postnatal day.

Since markers such as Calbindin and Oncomodulin are expressed in only striolar type I hair cells [34,35,29], we immunolabeled for Osteopontin for type I hair cells and for Annexin A4 and Mapt for type II hair cells [23]. We found that Osteopontin was expressed at the apical neck of type I hair cells with Tuj1^+^ calyces, while Annexin A4 and Mapt were expressed in the cell membrane of type II hair cells throughout the P30 utricle (Fig 2A–2D and 2K–2N, S2J and S2K Fig).

When examining HC^PG3^ in the P30 *Plp1-Tomato* utricles, we found that most traced hair cells expressed Annexin A4 (85.1 ± 25.8% of 117 and 94.9 ± 8.9% of 24 HC^PG3^ in the extrastriola and striola from 3 mice, respectively) (Fig 2C, S1A and S1B Fig, S3 Table). Similarly, most HC^PG8^ were Annexin A4^+^ (95.2 ± 8.2% of 24 and 100% ± 0% of 12 HC^PG8^ in extrastriola and striola from 3 mice, respectively) (Fig 2D, S2A and S2C Fig, S3 Table). We examined 95 Annexin A4^+^ HC^PG3^ and 35 Annexin A4^+^ HC^PG8^ and found that 69–79% were goblet-shaped, with other rare morphologic subtypes also present (Fig 2C and 2D, S2B, S2C and S2F Fig). Lastly, Annexin A4^+^ HC^PG8^ also expressed Mapt, a marker of mature type II hair cells [23] (82.4% of 17 extrastriolar cells and 76.5% of 17 striolar cells from 3 mice) (S2I–S2K Fig, S4 Table), suggesting that most postnatally generated hair cells from the Plp1 lineage have acquired a type II hair cell phenotype.

To verify their identity as type II hair cells, we probed HC^PG3^ and HC^PG8^ from P30 *Plp1-Tomato* utricles for both IKL, characteristic of type I hair cells, and IH non-specific cation currents [14,16]. The presence of IKL indicates a type I hair cell electrophysiological phenotype. To account for possible variations in measurements, we compared traced hair cells to untraced cells in a similar location within the sensory epithelium. To probe for IH we voltage-clamped hair cells at -69 mV and hyperpolarized between -74 and -156 mV for 400 ms and then back to -69 mV. Hair cells from each group (10/10 HC^PG3^, 9/9 HC^PG8^ and 5/5 untraced hair cells) displayed IH (Fig 2E–2G), with similar properties (peak conductance, half-activation and slope) measured in all three groups (Fig 2H–2J, S1 Data).

To assess whether postnatally generated hair cells can acquire features of type I hair cells, we immunostained for and found that a subset of HC^PG3^ and HC^PG8^ expressed Osteopontin (21.2 ± 32.6% of 87 and 19.3 ± 26.2% of 49 HC^PG3^ in the extrastriola and striola, respectively, from 3–4 mice, and 4.0 ± 6.8% of 81 and 1.7 ± 4.1% of 28 HC^PG8^ in the extrastriola and striola from 7–8 mice) (Fig 2M and 2N, S2D and S2E Fig, S3 Table). Among 24 Osteopontin^+^ HC^PG3^ and 2 HC^PG8^ examined, most (75–100%) displayed the classic amphora shape (Fig 2M and 2N, S2D, S2E and S2G Fig).

To further investigate whether postnatally generated hair cells exhibit type I hair cell electrophysiological properties, we probed and found no significant IKL in either HC^PG3^ or HC^PG8^ (*n =* 10 and 8 cells, respectively), suggesting that these groups do not contain HCs with a type I electrophysiological phenotype (Fig 2O, 2P and 2R). By contrast, IKL was detected in 75% of (9 of 12) untraced hair cells (Fig 2Q and 2R, S1 Data), with half-activation and slope comparable to those in mature type I hair cells (Fig 2S and 2T, S1 Data) [16].

The presence of IH and the lack of IKL in fate-mapped HC^PG3^ and HC^PG8^ suggest a non-type I hair cell phenotype. Although few postnatally generated hair cells displayed molecular and morphologic features of type I hair cells, none displayed such electrophysiological properties. This is consistent with the previous results that most postnatally generated hair cells displayed molecular and morphologic features representing a type II hair cell phenotype.

Of note, several conductances were not explored in detail, in part because they were not observed. Sodium conductances, often described in immature hair cells [14,36] were not observed in either traced or untraced cells, likely suggesting that all cells had matured past this developmental stage. A-type potassium conductances, which are often found in extrastriolar hair cells and used as a marker for maturation [14,37,16], were not observed in traced or untraced cells, likely indicating that we recorded from a location where this conductance was not observed.

In summary, these data indicate that Plp1^+^ supporting cells primarily give rise to type II hair cells, whereas few type I hair cells were generated in the postnatal period. In comparison, untraced Myosin7a^+^ hair cells, which likely represent hair cells that arose earlier than P8, more commonly expressed the type I hair cell marker Osteopontin (S3 Table) and the signature IKL at P30.

### Active presynaptic components in postnatally generated hair cells

**Fig 3:**
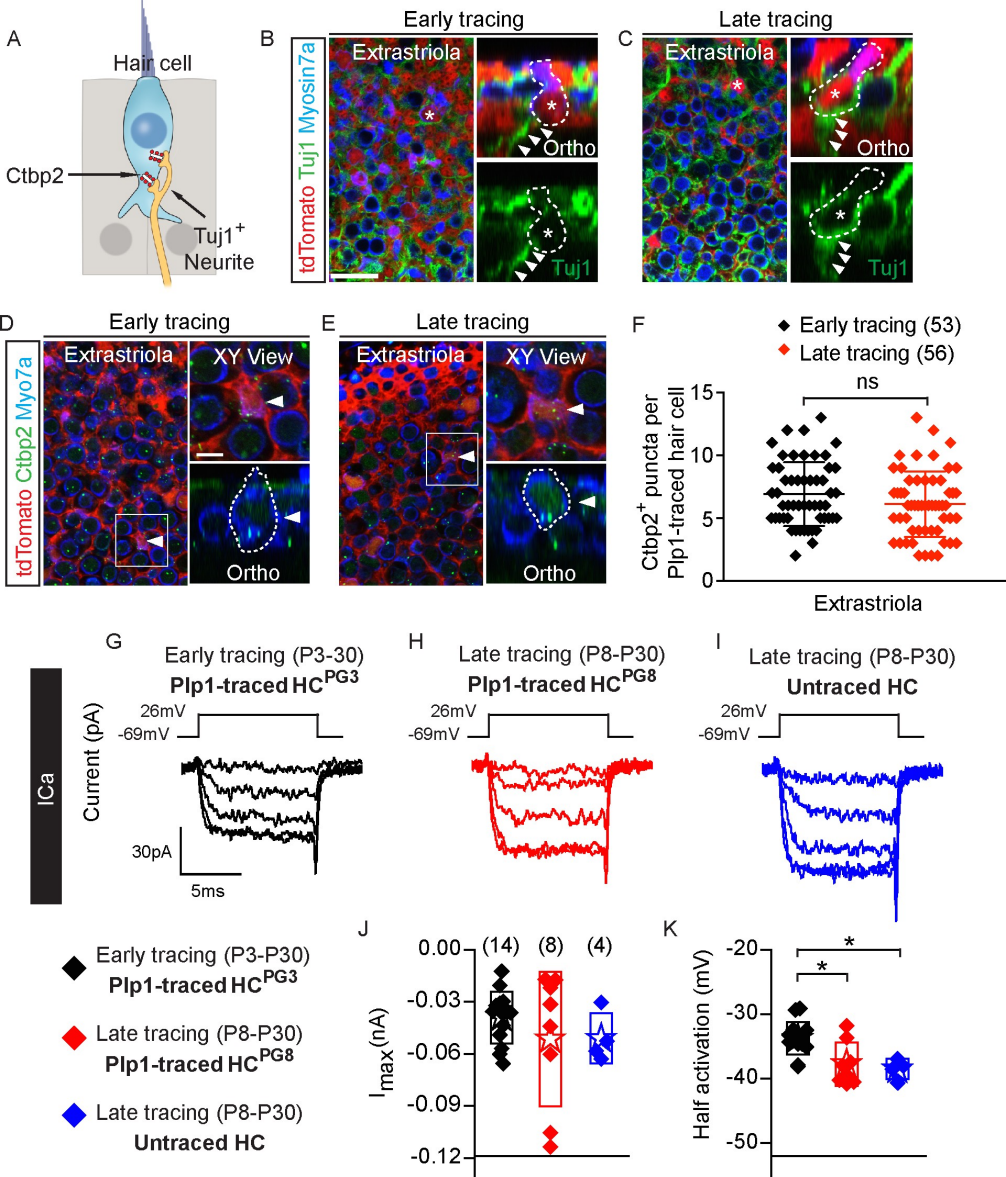
Synaptic properties of postnatally generated hair cells. A) Diagram illustrating hair cells with the presynaptic element Ctbp2 (red) and associated Tuj1^+^ vestibular neurites (beige). B-C) Representative confocal images of tdTomato^+^/Myosin7a^+^ hair cells (asterisks and dashed lines) from early and late tracing associated with Tuj1^+^ neurites (arrowheads in orthogonal views, *n =* 127 cells from 3 mice for early tracing and 73 cells from 9 mice for late tracing). D-E) Expression of Ctbp2 on the basolateral surfaces of traced hair cells (arrowhead). Shown are high magnification XY and orthogonal views of cells of interests in boxes (*n =* 53 cells from 3 mice for early tracing and 56 cells from 6 mice for late tracing). F) Quantification of Ctbp2^+^ puncta in tdTomato^+^/Myosin7a^+^ hair cells in the extrastriola. No significant difference was found between early and late tracing. G-I) Representative calcium currents from Plp1-traced HC^PG3^, HC^PG8^ and untraced hair cells. Calcium currents were isolated from voltage-clamped hair cells by replacing intracellular potassium with Cs and tetra-ethyl ammonium. Currents in response to the calcium current protocol were monitored and analyzed. J-K) Current-voltage plots were generated from peak current responses and maximal current and half activating voltage were extracted from these plots (*n =* 4–14 cells). Data shown as mean ± SD, compared using Student *t* tests and one-way ANOVA by Kruskal Wallis-Dunn's multiple comparison test. *p < 0.05. Scale bars: B-E) 20 μm, XY views in D-E) 5 μm. The underlying data can be found within S1 Data. HC^PG^, postnatally generated hair cell.

To determine whether postnatally generated hair cells are neurally integrated, we first examined innervation in P30 *Plp1-Tomato* utricles. All HC^PG3^ (*n =* 127 extrastriolar and 18 striolar hair cells from 3 mice) and HC^PG8^ (*n =* 73 extrastriolar and 18 striolar hair cells from 9 mice) were juxtaposed to Tuj1^+^ neurites (Fig 3A–3C, S3A and S3B Fig). However, neither HC^PG3^ nor HC^PG8^ showed any Tuj1^+^ calyx-shaped nerve terminals, supporting the notion that postnatally generated hair cells were primarily type II hair cells.

Ctbp2 is a major component of the hair cell presynaptic ribbon [38]. We found Ctbp2 expressed in all HC^PG3^ (*n =* 53 extrastriolar and 19 striolar hair cells from 3 mice) and HC^PG8^ (*n =* 56 extrastriolar and 25 striolar hair cells from 6 mice) examined (Fig 3A, 3D and 3E, S3C and S3D Fig). No significant difference was found in the number of Ctbp2^+^ puncta between HC^PG3^ and HC^PG8^ (Fig 3F, S3E Fig, S1 Data). In comparison to tdTomato-negative, untraced hair cells, the number of Ctbp2^+^ puncta of HC^PG3^ and HC^PG8^ also were also not significantly different (p > 0.05 for all).

We next examined whether HC^PG3^ and HC^PG8^ were capable of synaptic vesicle release. Calcium currents were isolated (Fig 3G–3I, S1 Data), current-voltage plots were generated from peak current responses, and the maximal current and half activating voltage were extracted from these plots (Fig 3J and 3K, S1 Data). Like untraced hair cells (4/4), all 14 HC^PG3^ and 8 HC^PG8^ displayed calcium current responses supportive of the potential for presynaptic activity. The presence of calcium currents is also supportive of traced cells taking on a hair cell phenotype. Slight differences in half-activation values may indicate different states of maturity and warrants further investigation.

To assess functional activity of presynaptic sites, vesicle fusion was monitored using a dual sine wave technique as previously described [39,40]. Three groups of hair cells (HC^PG3^, HC^PG8^, and untraced hair cells) were depolarized to the voltage of maximal calcium current for three seconds and changes in capacitance were monitored in real time (S3F–S3H Fig). We found that most (13/17 HC^PG3^, 7/8 HC^PG8^, 4/4 untraced hair cells) of the recorded cells had greater than 50 fF of capacitance change. Although quite variable, no differences in maximal release were observed among the groups (S3I and S3J Fig, S1 Data), supporting the conclusion that postnatally generated hair cells were presynaptically active. The presence of presynaptic activity also supports the conclusion that traced cells behave as hair cells.

### Immature stereociliary bundles and MET channels in postnatally generated hair cells

**Fig 4:**
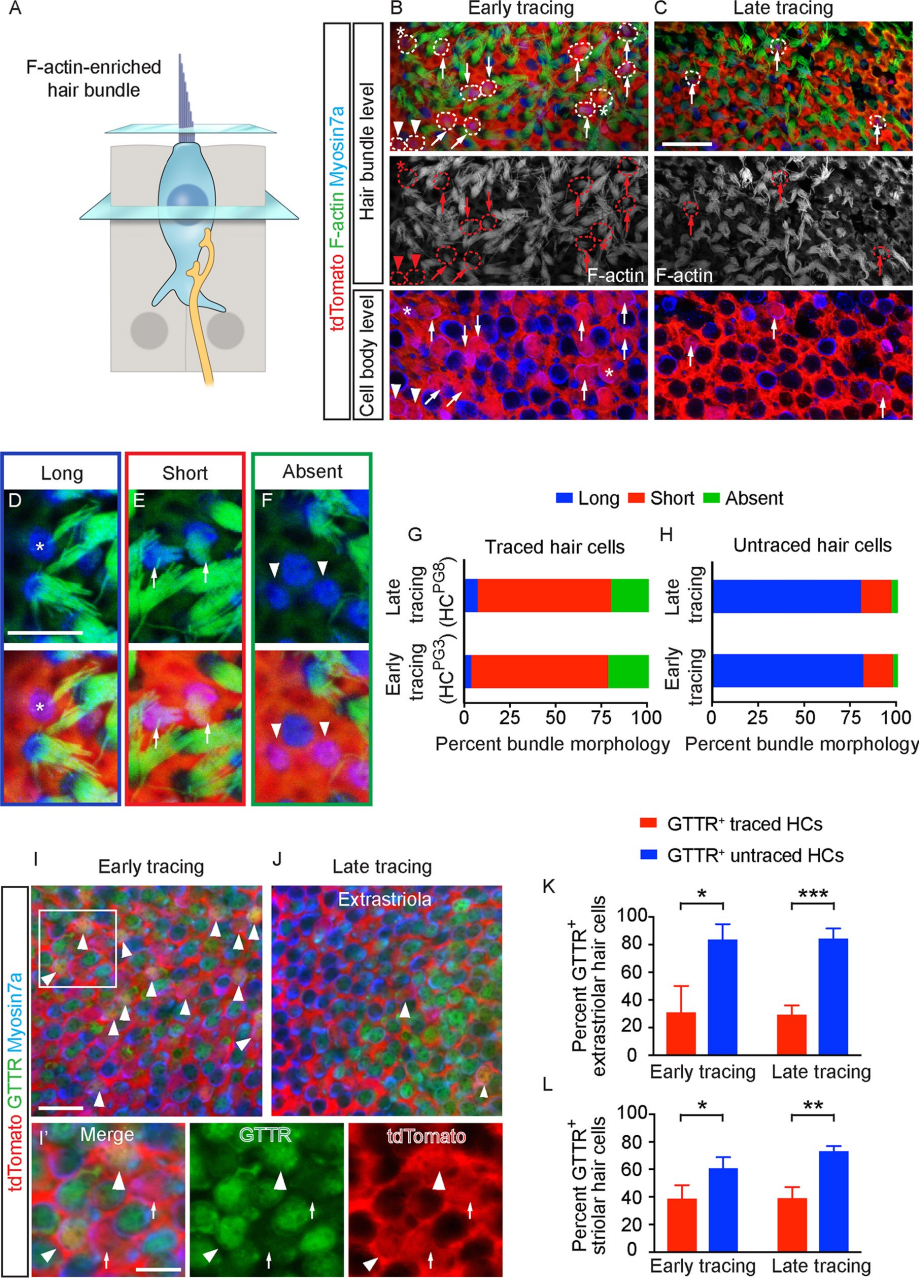
Bundle features of early and late postnatally generated hair cells. A) Diagram illustrating hair cell stereociliary bundles. B-C) tdTomato^+^/Myosin7a^+^ hair cells (dashed circles) added from early and late tracing with long (asterisks), short (arrows) and absent bundles (arrowheads) in both the hair bundle and cell body level. D-F) High magnification images of representative cells with long (D, asterisks), short (E, arrows) and absent bundles (F, arrowheads). G-H) Proportion of bundle morphology in traced and untraced hair cells. The majority of traced hair cells had short bundles from early (*n =* 127 HCs from 4 mice) and late tracing (*n =* 96 from 8 mice), whereas majority of untraced hair cells had long bundles (*n =* 849 HCs from 4 mice at P3, 794 HCs from 8 mice at P8). I-J) Representative confocal images of GTTR^+^/tdTomato^+^/Myosin7a^+^ (arrowhead) and GTTR^-^/tdTomato^+^/Myosin7a^+^ (arrows) hair cells from early and late tracing. I’) High magnification images of panel I. K-L) Significantly more untraced hair cells than traced hair cells were GTTR-labeled in both early (*n =* 117 and 48 traced HCs, 649 and 700 untraced HCs from 3 mice in the extrastriola and striola) and late tracing experiments (*n =* 30 and 16 traced HCs, 951 and 740 untraced HCs from 4 mice in the extrastriola and striola). Data shown as mean ± SD, compared using Student *t* tests. ***p < 0.001, **p < 0.01, *p < 0.05. Scale bars: B-C, I-J) 20 μm; D-F, I’) 10 μm. The underlying data can be found within S1 Data. P, postnatal day.

Another defining feature of hair cell maturation is the acquisition of stereociliary bundles and MET [14,41]. First, we marked these actin-rich structures using fluorescence-conjugated phalloidin and found that most HC^PG3^ and HC^PG8^ displayed either immature or no bundles (Fig 4A–4C). We categorized bundles based on established morphologic criteria (long, short and absent) [42] and discovered that most HC^PG3^ and HC^PG8^ displayed short or no stereocilia (74.8% and 21.3% of 127 HC^PG3^ from 4 mice and 72.9% and 19.8% of 96 HC^PG8^ from 8 mice; Fig 4D–4G, S1 Data). By contrast, more than 80% of untraced hair cells displayed mature-appearing (long) stereociliary bundles (*n =* 794–849 cells from 4–8 mice, Fig 4H, S1 Data). Immature bundles were similarly prevalent in HC^PG3^ and HC^PG8^ residing in the striolar and extrastriolar regions.

To test whether MET channels were functional in HC^PG3^ and HC^PG8^, we administered Texas-Red conjugated gentamicin (GTTR, 0.85 mg/ml for 1 hr), which enters hair cells via MET channels [43], to freshly harvested utricles. The presence of GTTR labeling indicates a population of MET channels that have a non-zero open probability. From early tracing (P3-P30) experiments, we found that GTTR labeled 83.7 ± 11.0% and 61.0 ± 7.8% of untraced hair cells in the extrastriola and striola of undamaged P30 utricles (*n =* 649 and 700 from 3 mice, Fig 4I–4L, S1 Data). This starkly contrasts the Plp1-traced hair cells, of which only 31.0 ± 19.0% and 38.9 ± 9.6% were GTTR-labeled (*n =* 117 and 48 in the extrastriola and striola regions from 3 mice). Similarly in the late tracing experiments (P8-P30), significantly more untraced hair cells were GTTR-labeled (84.2 ± 7.2% of 951 and 73.4 ± 3.4% of 740 hair cells in the extrastriola and striola from 4 mice) than traced hair cells (29.2 ± 6.7% of 30 and 39.2 ± 7.8% of 16 traced HCs in the extrastriola and striola from 4 mice, Fig 4I–4L, S1 Data). In summary, postnatally generated hair cells exhibit relatively underdeveloped bundles and MET channel activities in comparison to untraced hair cells.

### Hair cell regeneration and recovery of vestibular function after hair cell ablation

**Fig 5:**
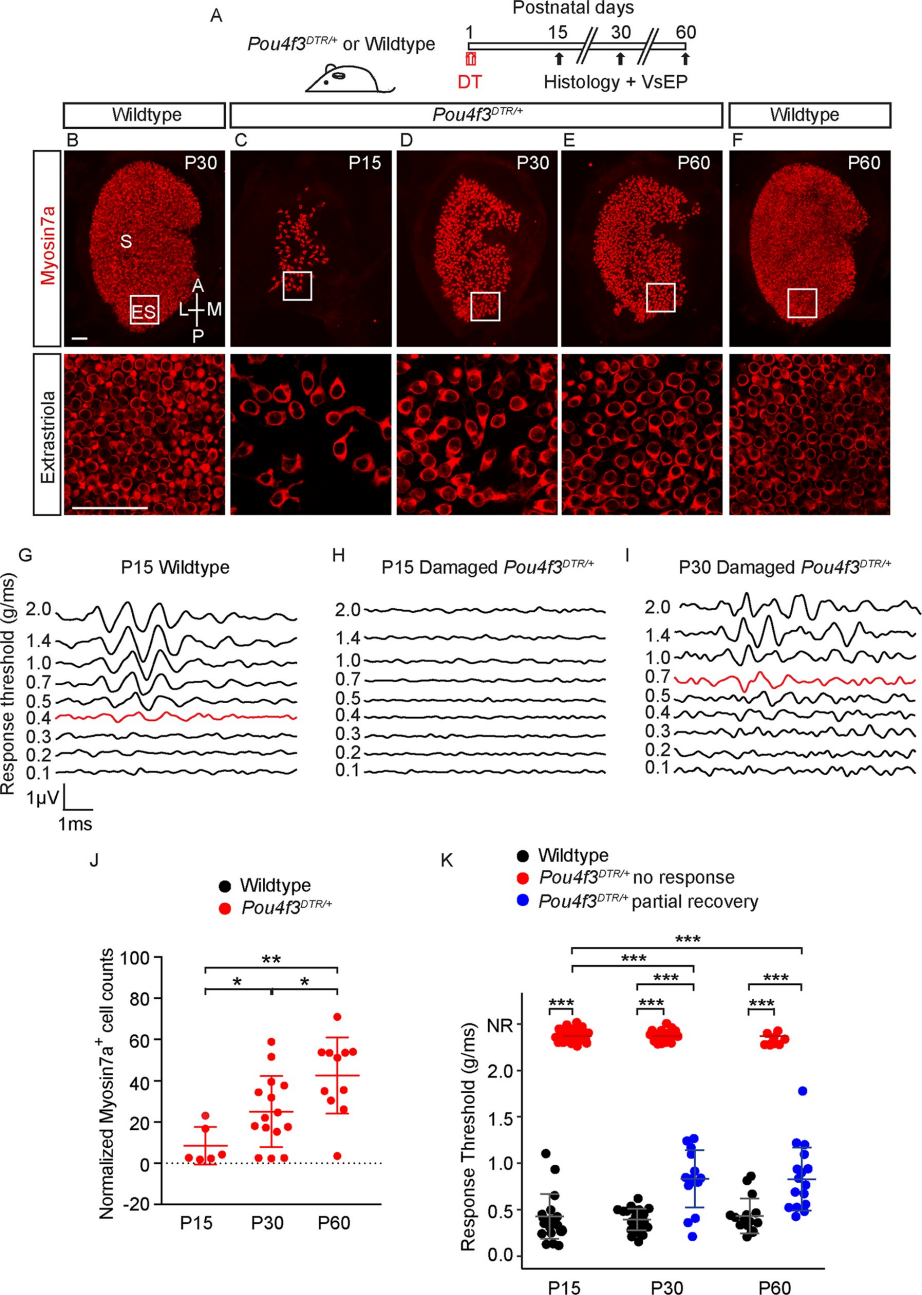
Hair cell number and vestibular function recovers after hair cell ablation. A) *Pou4f3*^*DTR/+*^ mice were treated with DT at P1 and examined at P15 and P30 and P60. B-F) After DT treatment, both the hair cell number and area of sensory epithelium decreased at P15, followed by a partial recovery at P30 and P60. G-I) Representative VsEP waveforms of normal responses of a P15 wild-type animal (0.4 g/ms threshold shown in red) (G), absent responses in a damaged P15 *Pou4f3*^*DTR/+*^ mouse (H), and an elevated response threshold in a damaged P30 *Pou4f3*^*DTR/+*^ mouse (0.7 g/ms threshold shown) (I). J) Normalized Myosin7a^+^ hair cell counts at P30 are significantly higher than P15 (*n =* 6 at P15 and 15 at P30). Damaged tissues normalized to age-matched controls. K) Many animals displayed partial VsEP thresholds recovery (blue dots) at P30 and P60 although some still showed no responses (red dots). Compared to P15, the average thresholds at P30 and P60 are significantly lower (*n =* 48 at P15, 39 at P30 and 27 at P60), but still significantly higher than age-matched controls (*n =* 20 at P15, 22 at P30, and 15 at P60). Data shown as mean ± SD, compared using Student *t* tests and one-way ANOVA by Tukey’s multiple comparison test. ***p < 0.001, **p < 0.01, *p < 0.05. Scale bars: 50 μm in B-F. The underlying data can be found within S1 Data. DT, diphtheria toxin; P, postnatal day; VsEP, vestibular-evoked potential.

We previously characterized hair cell regeneration in the neonatal utricle and found that both Plp1- and Lgr5-marked supporting cells contribute to the population of regenerated hair cells [9]. To examine the properties of regenerated hair cells (HC^R^), we ablated hair cells using the *Pou4f3*^*DTR/+*^ mouse [44], in which the hair cell promoter *Pou4f3* drives the expression of human diphtheria toxin receptor (DTR). Diphtheria toxin (DT) injection at P1 led to a loss of 91.5 ± 9.1% of hair cells two weeks later (P15, *n =* 6, Fig 5A, 5C and 5J, S4B and S4F and S4G Fig, S1 Data). At P30 and P60, hair cell numbers were restored to 25.1 ± 14.4% and 42.5 ± 18.5% of aged-matched controls, respectively (*n =* 15 from P30, 11 from P60, Fig 5B, 5D–5F and 5J, S4A and S4C–S4H Fig, S1 Data). Compared to P15, Myosin7a^+^ hair cell numbers at P30 and P60 in both the extrastriola and striola significantly increased, in line with previous results [9].

We next assessed function of the regenerating utricle by measuring vestibular-evoked potentials (VsEPs), which represent compound action potentials of the vestibular nerves and central relays as a result of a transient linear acceleration applied along the naso-occipital axis [45]. After DT-induced hair cell loss, all 51 P15 *Pou4f3*^*DTR/+*^ mice displayed elevated VsEP thresholds with 48 of 51 (94.1%) displaying no detectable responses, whereas undamaged P15 wild-type animals uniformly displayed robust VsEP thresholds (0.43 ± 0.24 g/ms, *n =* 20, Fig 5G, 5H and 5K, S1 Data). Among the 48 P15 animals that demonstrated no detectable VsEP thresholds, serial measurements showed that 35.9 and 59.3% regained thresholds at P30 and P60 with average response thresholds of 0.83 ± 0.31 g/ms and 0.83 ± 0.34 g/ms, respectively (*n =* 14 of 39 from P30 and 16 of 27 from P60). At these ages, the average responses significantly improved compared to P15 damaged animals (*p* < 0.001 for both, Fig 5I and 5K), but remained elevated compared to age-matched, undamaged animals (0.39 ± 0.12 g/ms and 0.43 ± 0.19 g/ms at P30 and P60, *n =* 22 and *n =* 15, Fig 5I and 5K, S1 Data). Thus, our data suggest that a subset of animals partially restore their ability to detect linear acceleration.

In support of this notion, VsEP latencies, which represent timing of the population of activated neurons, also significantly improved from P15 to P30 and P60 in DT-treated *Pou4f3*^*DTR/+*^ mice (S4I Fig). Similarly, VsEP amplitudes, which correlate with the size and synchrony of the population of activated neurons, also significantly improved from P15 to P30 and P60 of DT-treated *Pou4f3*^*DTR/+*^ mice (S4J Fig, S1 Data). We correlated the number of hair cells and VsEP thresholds and found the two to have a modest, inverse relationship (R^2^ = 0.62, S4K Fig, S1 Data). Together our data indicate that organ function is partially recovered following hair cell ablation in the neonatal utricle.

### Specialization of Plp1-traced regenerated hair cells into type II hair cells

**Fig 6:**
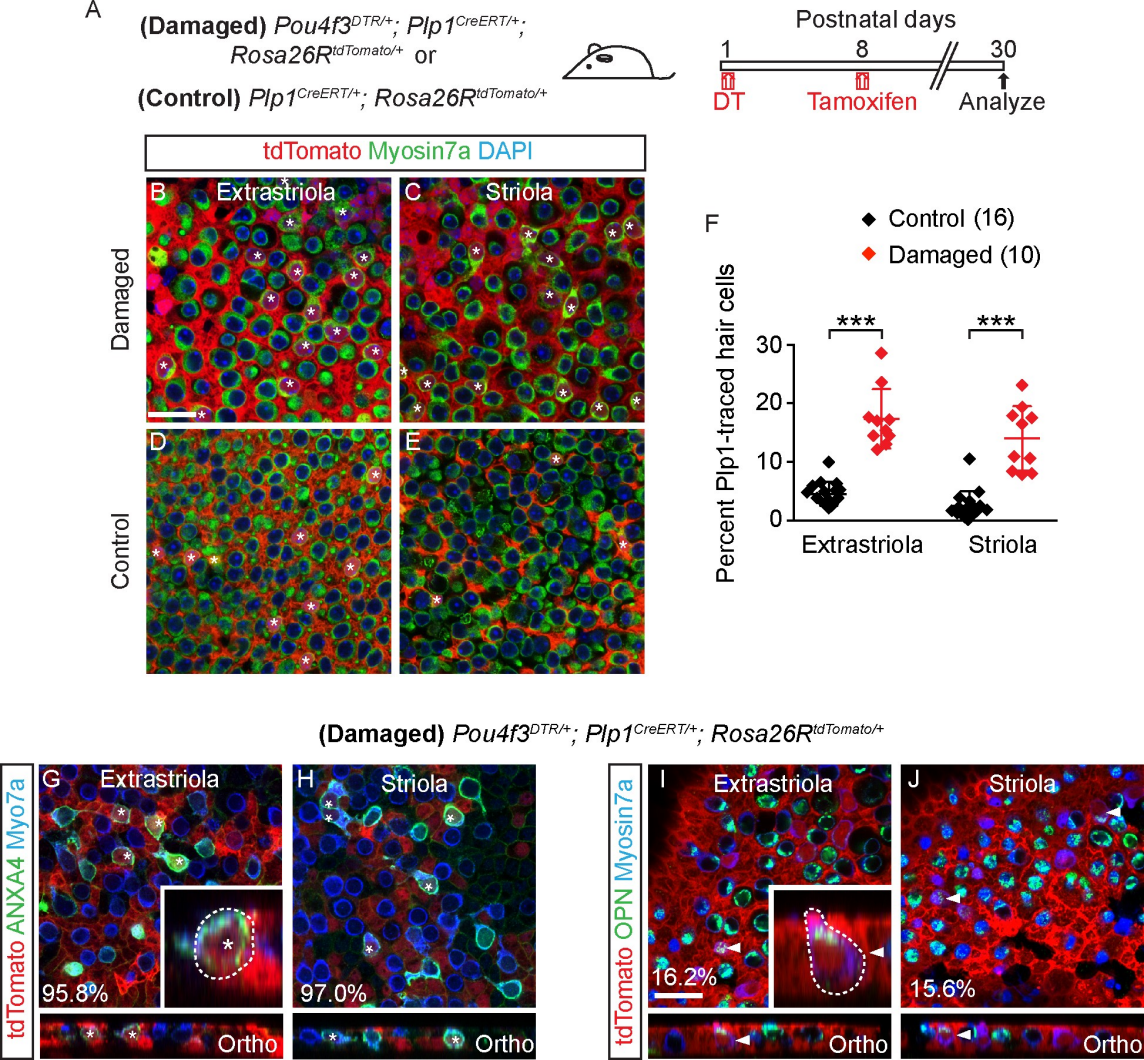
Plp1^+^ supporting cells regenerate hair cells after hair cell loss. A) *Pou4f3*^*DTR/+*^; *Plp1*^*CreERT/+*^*; Rosa26R*^*tdTomato/+*^ mice were treated with DT at P1, followed by tamoxifen at P8 to fate-map Plp1^+^ supporting cells. Organs were examined at P30. B-E) Damaged P30 utricles had more tdTomato^+^/Myosin7a^+^ hair cells (asterisks) than controls. F) Quantification shows that the percentage of tdTomato^+^/Myosin7a^+^ hair cells in damaged utricles (*n =* 10) was significantly higher than in undamaged controls (*n =* 16). G-H) Almost all traced, regenerated hair cells (asterisks) expressed the type II hair cell marker ANXA4 in both the extrastriola and striola. Inset shows orthogonal view of a traced, regenerated ANXA4^+^ hair cell with a short, round cell body and no basolateral processes. I-J) Occasionally traced, regenerated hair cells (arrowhead) expressed the type I hair cell marker OPN in both the extrastriola and striola. Inset shows orthogonal view of traced, regenerated OPN^+^ hair cells appearing pear-shaped, with a short neck. Data shown as mean ± SD and compared using Student *t* tests. ***p < 0.001. Scale bars: 20 μm. The underlying data can be found within S1 Data. ANXA4, Annexin A4; OPN, Osteopontin.

We next sought to examine the properties of regenerated hair cells (HC^R^) in the regenerating utricle in P30 *Pou4f3*^*DTR/+*^ mice. To identify HC^R^, we fate-mapped supporting cells in *Pou4f3*^*DTR/+*^*; Plp1*^*CreERT/+*^*; Rosa26R*^*tdTomato/+*^ (*Pou4f3-DTR; Plp1-Tomato*) mice. To minimize labeling of hair cells normally added postnatally, we administered tamoxifen at P8 (0.075 mg/g, IP) to fate-map Plp1^+^ supporting cells in the damaged utricle (Fig 6A, S5A–S5C Fig). As expected, we found significantly more fate-mapped, Myosin7a^+^ hair cells in both the extrastriolar and striolar regions (17.4 ± 5.1% and 14.0 ± 5.5%, *n =* 762–798 hair cells from 10 mice) than undamaged controls (4.6 ± 2.0% and 2.5 ± 2.5%, respectively, *p* < 0.001, *n* = 2,561–3,002 hair cells from 16 mice) (Fig 6B–6F, S1 Data). Consistent with published data [9], these results indicate that Plp1-marked supporting cells regenerated hair cells in the damaged neonatal mouse utricle.

In utricles from the P30 *Pou4f3-DTR; Plp1-Tomato* mice, almost all HC^R^ expressed Annexin A4 (95.8 ± 7.2% of 19 extrastriolar and 97.0 ± 5.2% of 22 striolar hair cells from 3 mice) and few expressed Osteopontin (16.2 ± 13.0% of 85 extrastriolar and 15.6 ± 12.0% of 83 striolar cells from 7 mice) (Fig 6G–6J, S3 Table). Most Annexin A4^+^ HC^R^, expressed the mature type II hair cell marker Mapt (76.5% of 17 extrastriolar cells and 58.8% of 17 striolar cells from 4 mice, S5F and S5G Fig, S4 Table). By contrast, the majority of untraced hair cells from damaged utricles expressed Osteopontin (68.0 ± 9.2% of 266 and 68.5 ± 11.4% of 292 untraced hair cells from 4 mice in the extrastriola and striola, respectively, S3 Table). Thus, the composition of untraced hair cells from damaged utricles, which likely represent surviving hair cells, is consistent with the previously reported ratios of type I and II hair cells in the striola and extrastriola (1.47 and 1.19) [46], whereas HC^R^ mainly displayed a type II hair cell phenotype. Unlike HC^PG3^ and HC^PG8^, many regenerated Annexin A4^+^ hair cells displayed short, rounded cell bodies without basolateral processes, while others displayed basolateral processes associated with classic type II hair cells (*n =* 37 cells from 3 mice) (Fig 6G and 6H, S5D Fig).

All regenerated Osteopontin^+^ hair cells examined (18/18) were also morphologically distinct from undamaged counterparts, appearing pear-shaped with short necks (Fig 6I and 6J, S5E Fig) and no amphora-shaped Osteopontin^+^, Plp1-traced hair cells were detected in the damaged organs. These results suggest that Plp1-traced HC^R^, like HC^PG3^ and HC^PG8^, preferentially acquire properties of type II hair cells and less commonly those of type I hair cells. However, many type II HC^R^ and all type I HC^R^ observed were morphologically distinct from HC^PG3^ and HC^PG8^.

**Fig 7:**
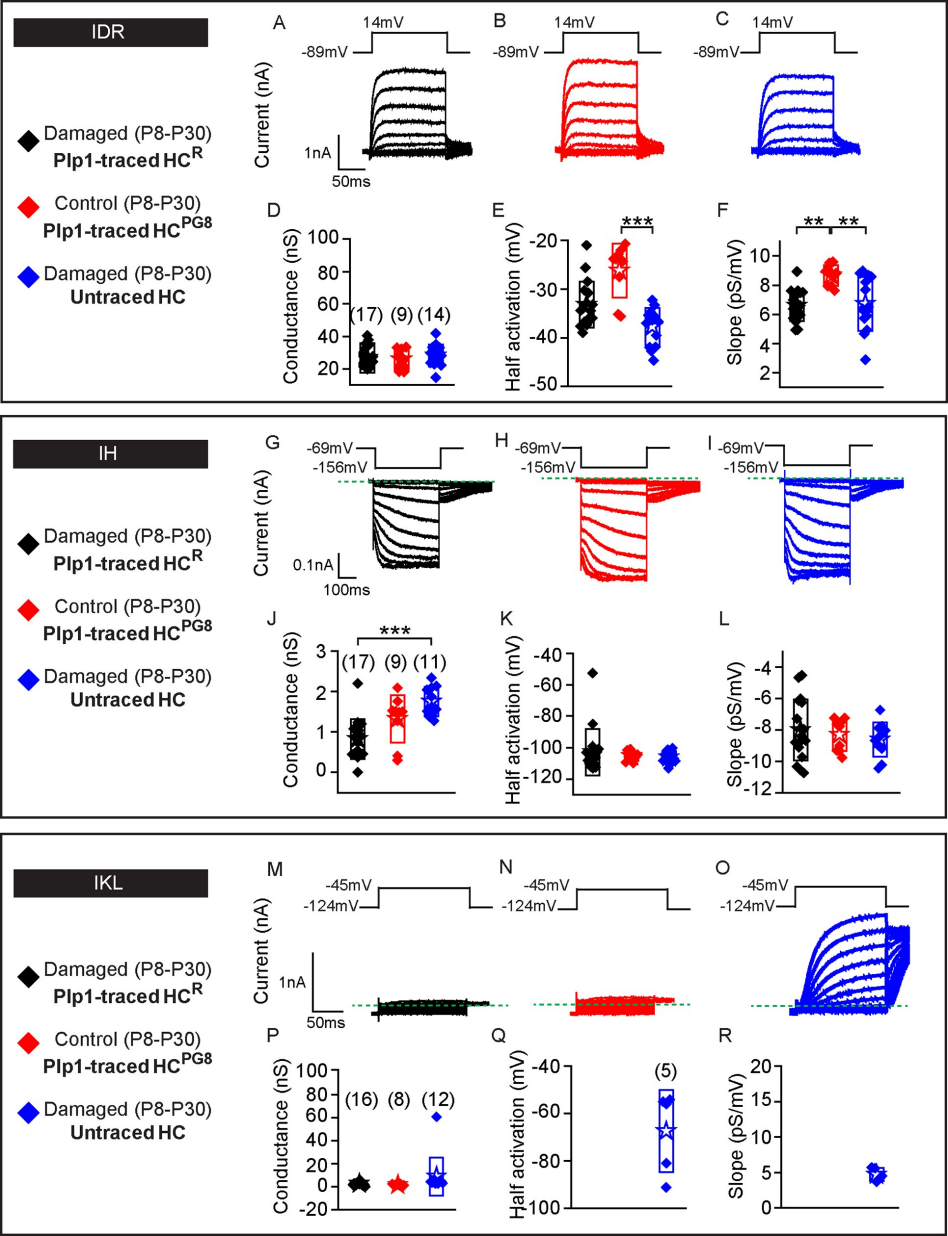
Plp1-traced, regenerated hair cells display electrophysiological features of type II hair cells. Three groups of hair cells were examined: 1) traced (black, HC^R^) and 2) untraced hair cells (blue) from P30 *Pou4f3*^*DTR/+*^*; Plp1*^*CreERT/+*^*; Rosa26R*^*tdTomato/+*^ mice treated with DT at P1 and tamoxifen at P8; and 3) traced hair cells (red, HC^PG8^) from *Plp1*^*CreERT/+*^*; Rosa26R*^*tdTomato/+*^ mice treated and tamoxifen at P8. A-C) Representative tracings of IDR from hair cells from each group (HC^R^, HC^PG8^ and untraced hair cells, *n =* 9–17 cells). D-F) All three groups demonstrate similar peak conductances but significant differences between half-activation and slopes. G-I) Tracings of IH measurements from HC^R^, HC^PG8^ and untraced hair cells (*n =* 9–17 cells). J-L) All three groups demonstrate similar peak conductances, half-activation, and slopes. M-O) IKL responses from untraced hair cells from the damaged utricles, but not HC^R^ or HC^PG8^ (*n =* 8–16 cells). P-R) Similar tail current and reversal potential analysis were performed and the resultant data for conductance, half-activation and slope for untraced hair cells are presented. Data shown as mean ± SD and compared using one-way ANOVA by Kruskal Wallis-Dunn's multiple comparison tests. Green dashed lines define zero current levels. ***p < 0.001, **p < 0.01. The underlying data can be found within S1 Data. HC^PG^, postnatally generated hair cell; P, postnatal day.

We next assessed the electrophysiological properties of Plp1-traced HC^R^ and untraced hair cells in P30 *Pou4f3-DTR; Plp1-Tomato* utricles (Fig 7A and 7C), and compared them to Plp1-traced HC^PG8^ from undamaged *Plp1-Tomato* utricles (Figs 2F and 7B). IDR were detected in all hair cells from the three groups (Fig 7A–7C, *n =* 17 HC^R^, 9 HC^PG8^ and 14 untraced hair cells, respectively) with similar corresponding maximal conductances (Fig 7D–7F, S1 Data), despite differences in half-activation and slope. Cell capacitance was used as a proxy for cell size, which serves as a marker for hair cell maturation. Traced hair cells, like untraced hair cells, displayed capacitance measurements similar to those of postnatal utricular hair cells (S2 Table) [14]. Similarly resting potential data varies with cell maturity and measured values suggest that HC^R^ were at least as mature as postnatal hair cells previously reported. Although the absolute level of maturity cannot be deduced from these parameters, these data suggests that traced hair cells match untraced hair cells.

Next, we probed for and found IH in all three groups of hair cells (17/17 HC^R^, 9/9 HC^PG8^ and 11/11 untraced hair cells) (Fig 7G–7I). Although hair cells from the three groups showed comparable voltages of half-activation and voltage dependence (slope), conductances of HC^R^ were lower than those of untraced hair cells suggesting fewer channels are present in these newly formed cells (Fig 7J–7L, S1 Data) and perhaps indicating a less mature population. Conversely, IKL was not detected in any Plp1-traced HC^R^ or HC^PG8^ (0/16 and 0/8, respectively, Fig 7M and 7N) but was detected in 5/12 untraced hair cells examined (Fig 7O–7R, S1 Data). These data indicate that Plp1-traced HC^R^ displayed electrophysiological properties representative of non-type I hair cells, and together with the molecular markers support a type II hair cell phenotype.

### Regenerated hair cells display synaptic elements but immature bundles

**Fig 8:**
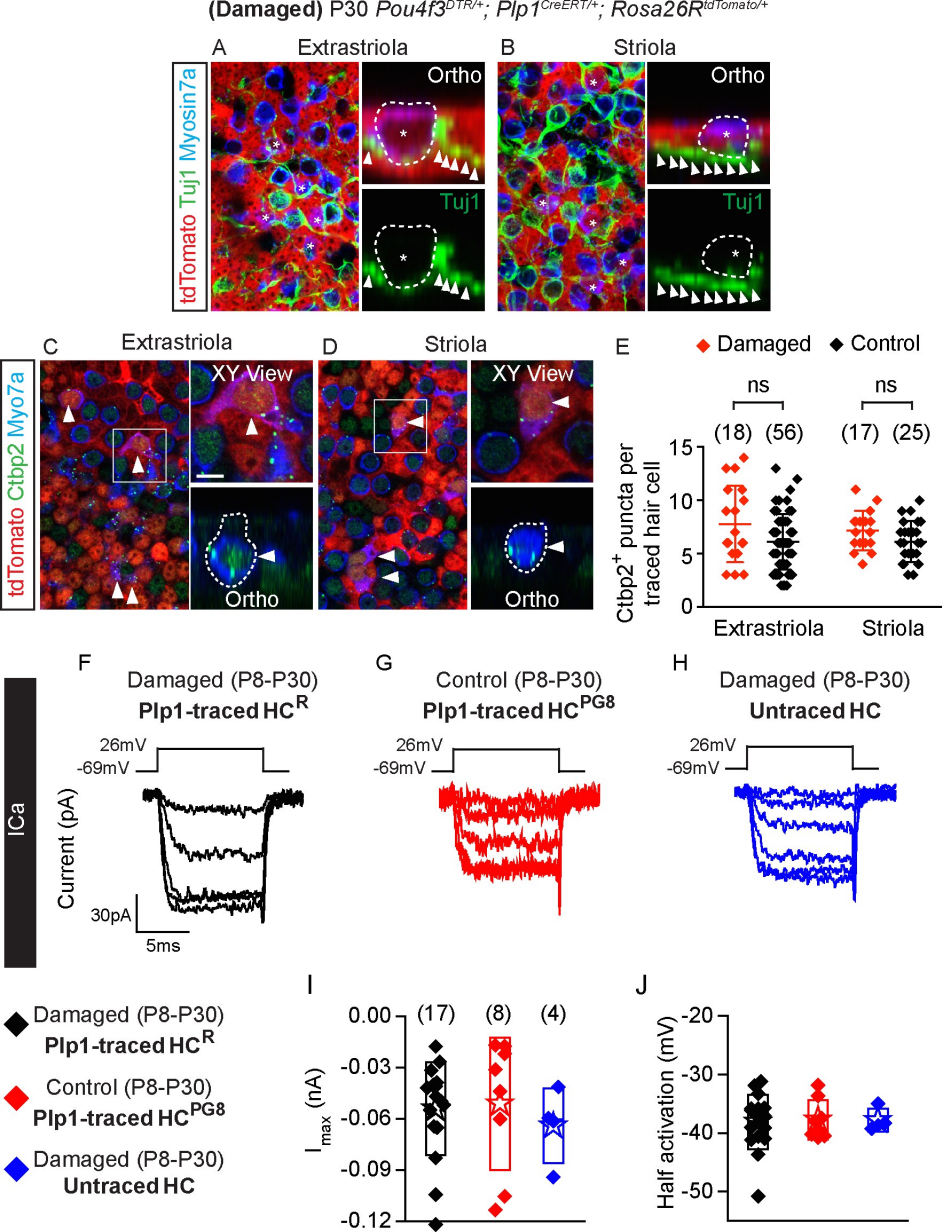
Synaptic properties of Plp1-traced, regenerated hair cells. A-B) Representative images of regenerated tdTomato^+^/Myosin7a^+^ hair cells (asterisks) with associated with Tuj1^+^ neural elements (arrowheads) in the extrastriola and striola (*n =* 106 cells from 5 mice). C-D) All Plp1-traced hair cells examined expressed Ctbp2 on the basolateral surfaces (arrowheads, *n =* 35 cells from 3 mice). E) Quantification of Ctbp2^+^ puncta in traced hair cells in the extrastriola and striola. No significant difference was found between hair cells from control and damaged utricles (*n =* 56 extrastriolar and 25 striolar hair cells from 6 control mice utricles, *n =* 18 extrastriolar and 17 striolar hair cells from 3 damaged mice utricles). F-H) Representative calcium currents from traced hair cells (HC^R^ from damaged utricles and HC^PG8^ from undamaged utricles) and untraced hair cells. Calcium currents were isolated using methods described in Fig 3. I-J) Peak current responses and maximal current and half activating voltage were not statistically different among groups (*n =* 4–17 cells). Data shown as mean ± SD, compared using Student *t* tests and one-way ANOVA by Kruskal Wallis-Dunn's multiple comparison tests. Scale bars: A-D) 20 μm, XY view) 5 μm. The underlying data can be found within S1 Data. HC^PG^, postnatally generated hair cell.

We next examined HC^R^ for formation of presynaptic terminals and innervation by immunostaining for Ctbp2 and Tuj1 in P30 *Pou4f3-DTR; Plp1-Tomato* utricles. In all HC^R^ examined (*n =* 106 cells in both extrastriola and striola from 5 mice) we found adjacent Tuj1^+^ neurites with no calyces observed (Fig 8A and 8B). HC^R^ also uniformly expressed Ctbp2, with the number of Ctbp2^+^ puncta similar to those of HC^PG8^ (combined from both extrastriola and striola, *n =* 81 and 35 cells from 6 control and 3 damaged mice, Fig 8C–8E, S1 Data). These data suggest that HC^R^ had presynaptic machinery and innervation consistent with a hair cell phenotype.

To further characterize HC^R^ for the presence of functional presynaptic elements, we assessed calcium currents and monitored presynaptic vesicle fusion via capacitance changes in response to depolarization in HC^R^ (Fig 8F–8H, S5H–S5J Fig, S1 Data). Like HC^PG8^ (8/8) and untraced hair cells (4/4) from the damaged organ, calcium currents were detected in all HC^R^ (17/17) examined and found to be of comparable sizes and voltage-dependence (Fig 8I and 8J, S1 Data). The presence of calcium currents supports a hair cell phenotype and the potential for presynaptic activity. Capacitance measurements showed calcium driven changes indicative of vesicle release in almost all HC^R^ (14/15, 7/8 HC^PG8^ and 3/3 untraced hair cells, S5K and S5L Fig, S1 Data), suggesting that the morphologically identified presynaptic elements were likely functional.

**Fig 9:**
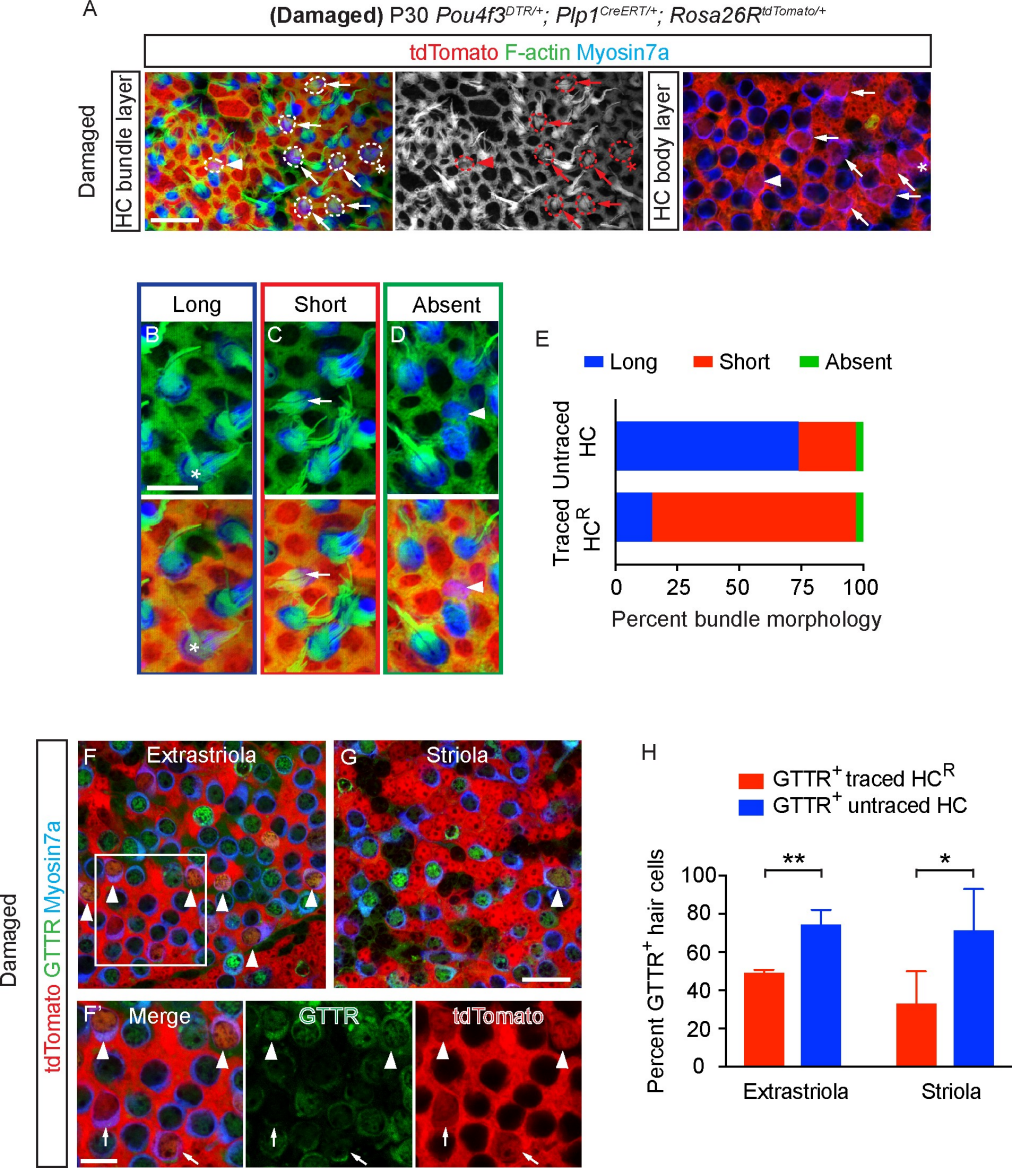
Regenerated hair cells display immature stereociliary bundles. A) Regenerated traced hair cells (dashed circles) with long (asterisks), short (arrows) and absent bundles (arrowheads). Shown are images taken at the focal planes of hair bundle and hair cell body. B-D) Representative high magnification images of hair cells with long (B) (asterisk), short (C) (arrow) and absent bundles (D) (arrowhead). E) Proportion of traced and untraced hair cells displaying the above bundle morphology. Most regenerated hair cells had short bundles (124 cells from 6 damaged mice), whereas most untraced hair cells had long bundles (617 cells from 6 damaged mice). F-G) Representative confocal images of GTTR^+^/tdTomato^+^/Myosin7a^+^ (arrowhead) and GTTR^-^/tdTomato^+^/Myosin7a^+^ hair cells (arrows) in damaged utricles. F’) High magnification images of panel F. H) Compared to traced hair cells, significantly more untraced hair cells were GTTR-labeled in the damaged utricles (*n =* 25 and 11 traced HCs, 172 and 148 untraced HCs from 4 mice in the extrastriola and striola). Data shown as mean ± SD, compared using Student *t* tests. **p < 0.01, *p < 0.05. Scale bars: A, F-G) 20 μm; B-D, F’) 10 μm. The underlying data can be found within S1 Data.

Next, we examined the bundles of HC^R^ via labeling with fluorescence-conjugated phalloidin (Fig 9A) and found that they, like HC^PG8^, mostly displayed immature stereociliary bundles: 14.5% long, 82.3% short and 7.3% absent stereocilia (*n =* 124 cells in both extrastriola and striola from 6 mice, Fig 9A–9E, S1 Data). By contrast, most untraced hair cells in damaged tissues displayed long bundles (*n =* 617 cells in both extrastriola and striola from 6 mice in damaged utricles, Fig 9E, S1 Data).

After adding GTTR to acutely isolated damaged/regenerated P30 utricles, we found that GTTR labeled 74.7 ± 7.4% and 71.7 ± 21.4%% of untraced hair cells (*n =* 172 and 148 untraced HCs in the extrastriola and striola, respectively, Fig 9F–9H, S1 Data) versus 49.3 ± 21.5% and 33.3 ± 21.5% of traced hair cells (25 and 11 traced HCs in the extrastriola and striola from 4 mice). These data suggest that fewer traced, regenerated hair cells have open MET channels (with non-zero open probability) than untraced hair cells. Taken together, these results suggest that Plp1-traced regenerated hair cells display some somatic properties of type II hair cells, yet immature stereociliary bundles and MET.

### Damage-activated Lgr5^+^ supporting cells regenerate type I and II hair cells

**Fig 10:**
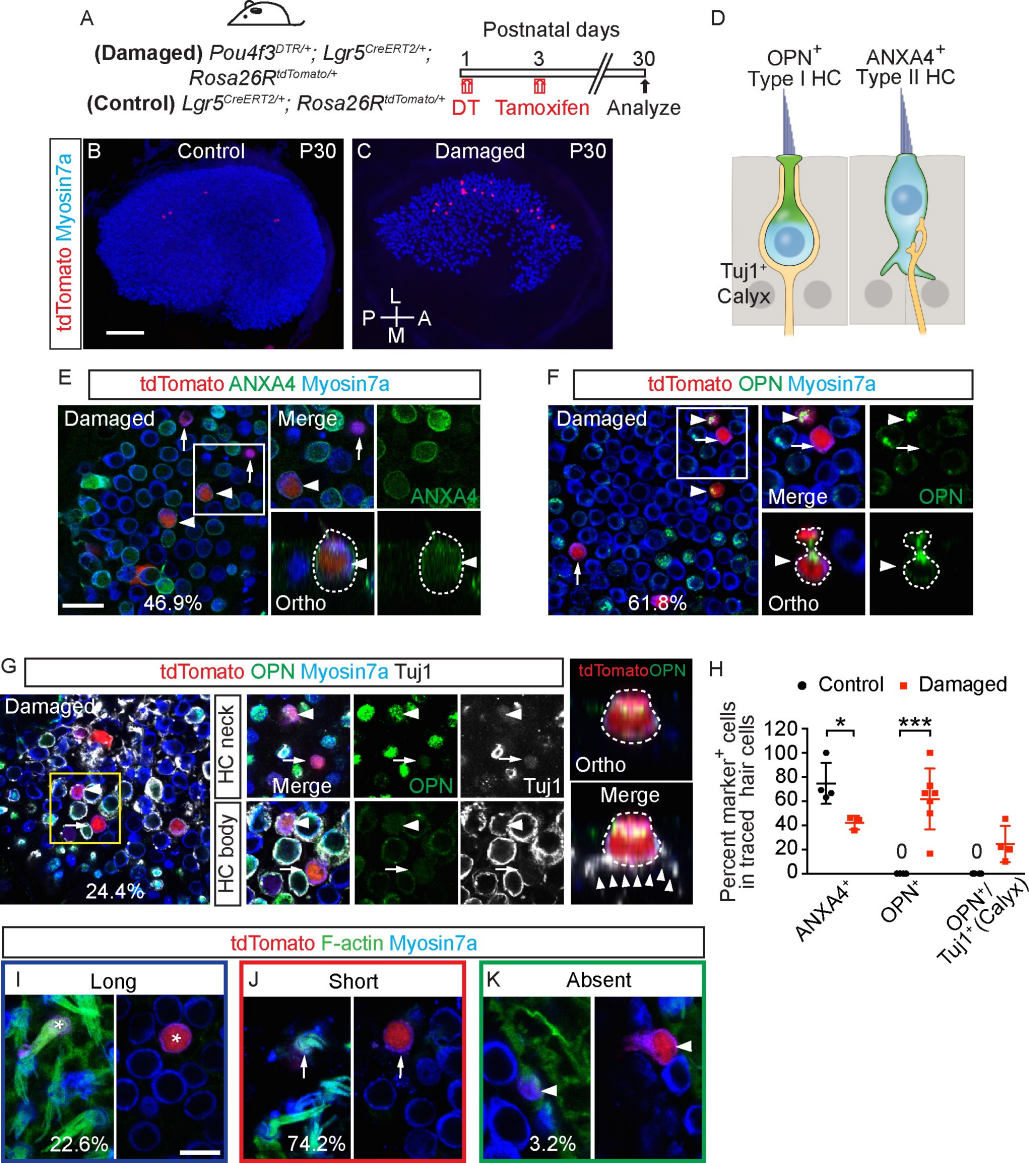
Lgr5^+^ supporting cells regenerate type I and type II hair cells. A) *Pou4f3*^*DTR/+*^; *Lgr5*^*CreERT2/+*^*; Rosa26R*^*tdTomato/+*^ and *Lgr5*^*CreERT2/+*^*; Rosa26R*^*tdTomato/+*^ mice were treated with DT at P1, followed by tamoxifen at P3 to fate-map Lgr5^+^ supporting cells. Organs were examined at P30. B-C) Whole mount preparation of P30 control and damaged utricles. Traced cells primarily occupied the presumed striolar region of the damaged organ. D) Diagram illustrating type I and II hair cells, the former of which are endowed with Tuj1^+^ calyx. E) Representative images of ANXA4^+^ (arrowhead) and ANXA4^-^ (arrow) traced hair cells in the P30 damaged utricle. 46.9% of Lgr5-traced hair cells expressed ANXA4 (*n =* 36 cells from 3 mice). F) Representative images of OPN^+^ (arrowhead) and OPN^-^ (arrow) traced hair cells in the P30 damaged utricle. 61.8% of Lgr5-traced hair cells were OPN^+^ (*n =* 71 cells from 7 mice). G) In the P30 damaged utricle, Lgr5-traced hair cells expressed OPN (arrowhead) and were surrounded by Tuj1^+^ calyx (arrowhead, 24.4% of Lgr5-traced hair cells, *n =* 40 cells from 4 mice), or were OPN^-^ and innervated by Tuj1^+^ boutons (arrow). Representative orthogonal view shows round, pear-shaped, regenerated hair cells with OPN^+^, short neck and Tuj1^+^ calyx (arrowheads). H) Compared to undamaged controls, the damages utricles had significantly fewer ANXA4^+^ traced HC^R^ and more OPN^+^ and OPN^+^/Tuj1^+^ (Calyx) traced HC^R^. No OPN^+^ and OPN^+^/Tuj1^+^ (Calyx) traced hair cells were seen in the undamaged utricle. Data shown as mean ± SD, compared using Student *t* tests. (I-K) Representative high magnification images of traced hair cells with long (I) (asterisk), short (J) (arrow) and absent bundles (K) (arrowhead). ***p < 0.001, *p < 0.05. Scale bars: B-C) 100 μm; E-G) 20 μm. (I-K) 10 μm. The underlying data can be found within S1 Data. ANXA4, Annexin A4; OPN, Osteopontin.

Thus far, we have shown that the Plp1-lineage supporting cells primarily regenerated hair cells displaying a type II hair cell phenotype. Previously, we found that damage activates Lgr5^+^ supporting cells in the striolar region to regenerate both type I and II hair cells, which were defined only by expression of Calbindin, Sox2, and the presence of Tuj1^+^ calyx nerve terminals [9]. To further investigate the properties of HC^R^ from the Lgr5^+^ lineage, we fate-mapped Lgr5^+^ supporting cells in *Pou4f3*^*DTR/+*^*; Lgr5*^*CreERT2/+*^*; Rosa26R*^*tdTomato/+*^ (*Pou4f3-DTR; Lgr5-Tomato*) mice (DT at P1 to induce hair cell loss and tamoxifen at P3 to trace Lgr5^+^ cells) (Fig 10A). The latter time point (P3) was selected because Lgr5 is transiently up-regulated in supporting cells [9]. DT-induced damage led to significantly more tdTomato^+^, Myosin7a^+^ hair cells than in undamaged controls at P30 (82.6 ± 11.0% and 65.4 ± 12.7%, *p* < 0.01, n = 135–164 cells from 9–10 mice, Fig 10B and 10C). Lgr5-traced HC^R^ primarily resided in the striola, and more often expressed the type I marker Osteopontin (61.8 ± 25.2% of 71 cells from 7 mice) than the type II marker Annexin A4 (46.9 ± 2.8% of 36 cells from 3 mice, Fig 10D–10F, S3 Table). Moreover, 24.4 ± 15.0% of Lgr5-traced HC^R^ displayed Tuj1^+^ calyces (*n =* 40 cells from 4 mice, Fig 10G, S6E Fig, S3 Table). In undamaged controls, many Lgr5-traced hair cells expressed Annexin A4 (76.6 ± 20.3%, *n =* 57 cells from 4 mice), but none expressed Osteopontin (*n =* 65 cells from 6 mice) or displayed Tuj1^+^ calyces (*n =* 26 cells from 3 mice) (Fig 10H, S6A–S6D Fig, S3 Table, S1 Data). Similar to Plp1-traced hair cells, most Lgr5-traced hair cells displayed short or no bundles (74.2% and 3.2% of 31 cells from 6 mice) and 22.6% of them displayed long bundle (Fig 10I–10K). These data suggest that Lgr5-traced supporting cells, in contrast to Plp1-traced supporting cells, can give rise to both type I and type II hair cells, although mostly presumably nonfunctional given the presence of immature stereociliary bundles.

**Fig 11:**
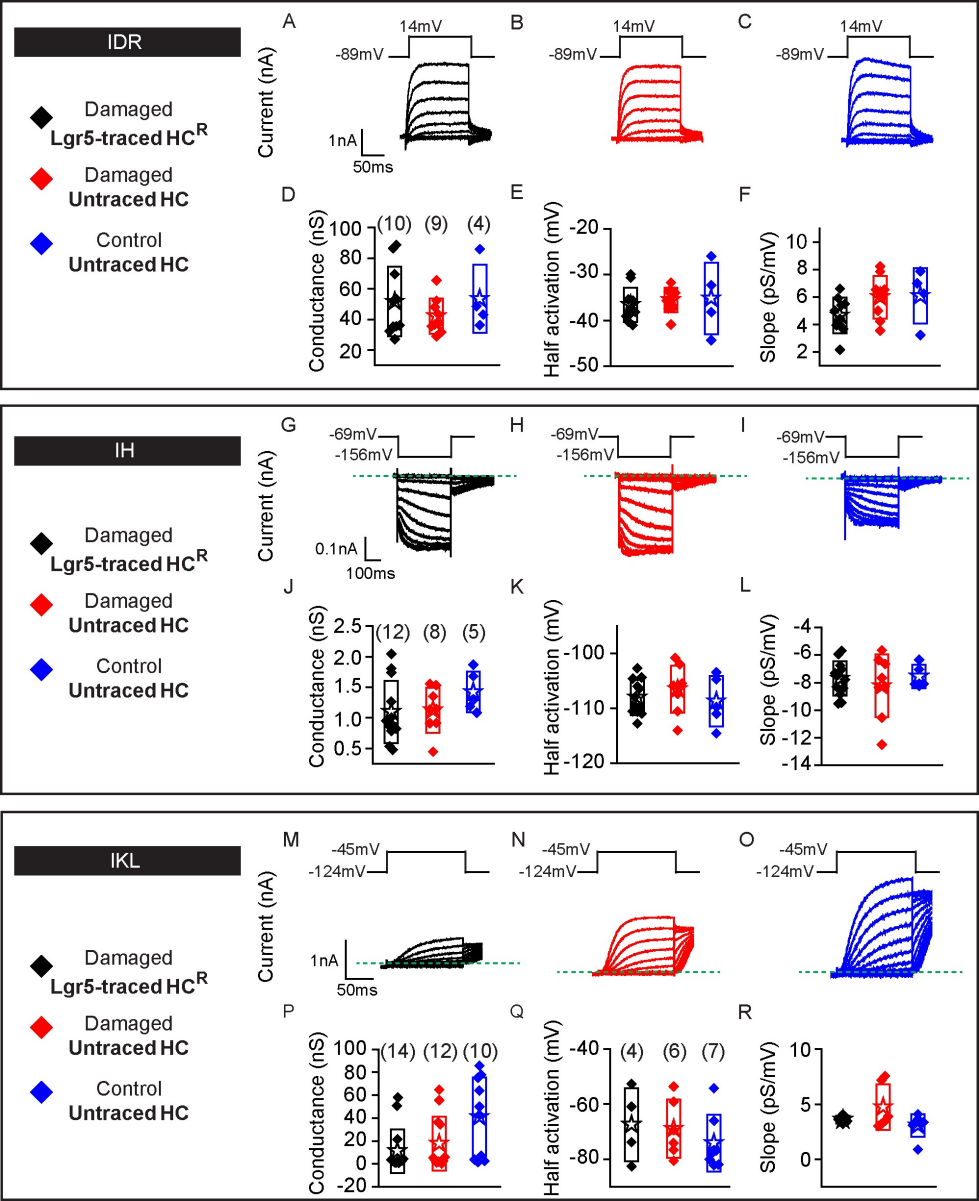
Lgr5-traced, regenerated hair cells display electrophysiological features of type II and type I hair cells. Three groups of hair cells were examined: 1) traced (black, HC^R^) and 2) untraced (red) hair cells from P30 *Pou4f3*^*DTR/+*^; *Lgr5*^*CreERT2/+*^*; Rosa26R*^*tdTomato/+*^ mice treated with DT at P1 and tamoxifen at P3; and untraced (blue) hair cells from *Lgr5*^*CreERT2/+*^*; Rosa26R*^*tdTomato/+*^ mice treated with tamoxifen at P3. A-C) Representative tracings of IDR from hair cells from each group (HC^R^, untraced hair cells from damage and controls, *n =* 4–10 cells). D-F) All three groups demonstrate similar peak conductances, half-activation and slopes. G-I) Tracings of IH measurements from HC^R^, and untraced hair cells from damaged and undamaged organs (*n =* 5–12 cells). J-L) All three groups demonstrate similar electrophysiological properties: peak conductances, half-activation and slopes. M-O) IKL responses from HC^R^, and untraced hair cells from damaged and undamaged organs (*n =* 10–14 cells). P-R) Conductance, half-activation and slopes were no different among the three groups. Data shown as mean ± SD, compared using one-way ANOVA by Kruskal Wallis-Dunn's multiple comparison tests. Green dashed lines define zero current levels. The underlying data can be found within S1 Data.

We next compared the electrophysiological properties of Lgr5-traced HC^R^ which demonstrated comparable gDR and gH and voltage-dependent properties relative to control, untraced hair cells in damaged and undamaged P30 utricles (*n =* 4–12 cells) (Fig 11A–11L, S1 Data). Remarkably, gKL, a marker for type I hair cells, were prominent in 4 of 14 patched, Lgr5-traced HC^R^ (as compared to 6 of 12 untraced hair cells from damaged utricles and 7 of 10 untraced hair cells from undamaged utricles) (Fig 11M–11R, S1 Data). Among these 4 Lgr5-traced HC^R^ with prominent gKL, three demonstrated large conductances (>20 nS) typically detected in mature type I hair cells [47,16].

Calcium currents and capacitance were measured in Lgr5-traced HC^R^ to assess for presynaptic activity (S6F–S6H Fig). In comparison to untraced hair cells in damaged and undamaged organs, calcium currents in HC^R^ were of comparable size and voltage-dependence, suggesting that these cells were presynaptically active and consistent with a hair cell phenotype (*n =* 5–8 cells) (S6I and S6J Fig, S1 Data). Capacitance changes were found in ~50% of HC^R^, with no differences in maximal release properties among groups (*n =* 4–7 cells) (S6K–S6O Fig, S1 Data), implying the presence of vesicle release in a subset of Lgr5-derived, regenerated hair cells. Together, these results indicate that Lgr5^+^ supporting cells can regenerate hair cells with either a type I or II hair cell phenotype as defined by molecular and electrophysiological features.

## Discussion

Mechanoreceptors are critical for hearing and balance functions. In mammals, the cochlea lacks the ability to regenerate, and vestibular hair cells are replenished in a limited fashion. As stereociliary bundles house the apparatus for MET, many prior studies examining regenerated hair cells or newly generated hair cells in ex vivo conditions have relied on assessing bundle morphology to determine hair cell maturity [5,48,20]. In this study, we show that Plp1-traced postnatally generated and regenerated hair cells exhibit several morphologic, molecular and electrophysiological properties of type II hair cells, though they continue to display largely immature hair bundles. Lgr5^+^ supporting cell-derived hair cells display underdeveloped hair bundles as well, but contain morphological, molecular and electrophysiological properties of both type I and type II hair cells, confirming and extending previous findings [9]. Our study indicates that hair cells postnatally re/generated from Plp1^+^ and Lgr5^+^ supporting cells can reach a quasi-mature level with regards to somatic properties, although the apical stereociliary bundles and MET machinery appear largely underdeveloped, suggesting that complex and partially distinct mechanisms are involved.

### Discrepancy between bundle and somatic features in postnatally generated and regenerated hair cells

In the developing utricle, hair cells are first specified between E11.5–12.5 with a second wave appearing at approximately E15, with hair cell addition presumably mainly in the peripheral regions of the sensory epithelia [49,11,50]. Hair cell bundles first emerge at E13.5 and remain relatively uniform in height throughout the central region at E15, when hair cells gain mechanosensitivity [49,13,14,51]. Géléoc and colleagues reported that a delayed rectifier potassium conductance (gDR) and a fast inward rectifier potassium conductance (gK1) begin to appear around E14-E15 and continue to be present into the postnatal period [14]. The transient expression of sodium conductances (gNA) in immature hair cell has also been described [14,36]. In addition, cell size based on capacitance measurements and resting membrane potentials change as hair cells mature from the embryonic to postnatal periods [14].

Transmission electron microscopy shows that afferent neurites appear as early as E13, and bouton synaptic contacts between hair cells and afferent nerve fibers form at E15 [52]. However, calyceal afferent endings, which are uniquely coupled to type I hair cells, only begin to take shape several days before birth and continue developing and maturing during the first postnatal month [16,53]. Moreover, embryonic hair cells acquire synaptic elements and display calcium-dependent exocytotic activities, which have been reported to mature postnatally although the time course is largely unclear [54,55].

Lastly, the type I hair cell-specific delayed rectifier potassium conductance (gKL) [56–58,16] is first detected at E18, shortly before birth. The delayed, inward rectifier H-type conductance (gH) is also detected in embryonic hair cells and both types of hair cells in the postnatal period [16]. In the mature utricle, the inward rectifier potassium conductances (gKIR) are expressed in type II hair cells [14].

**Fig 12:**
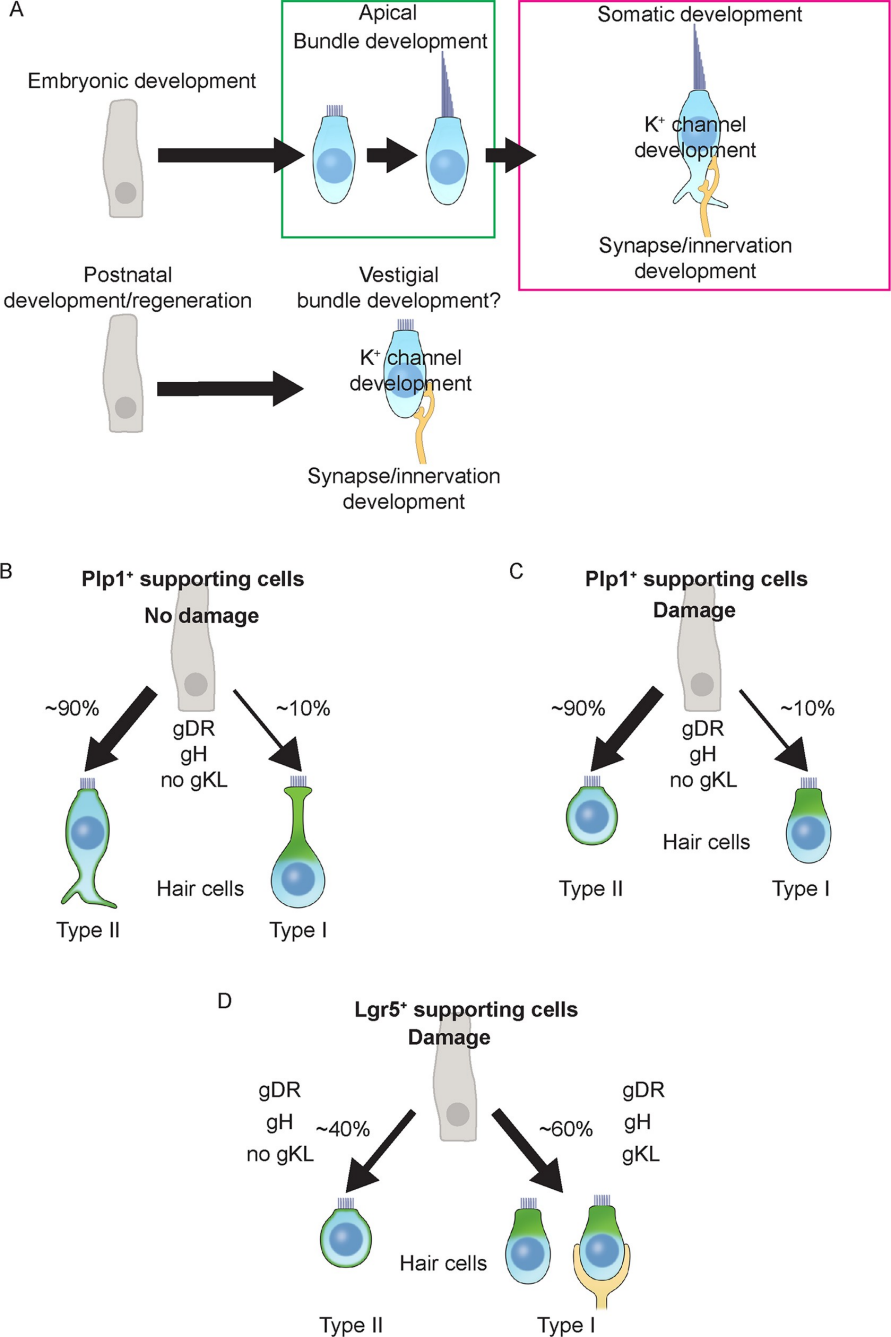
Maturation pathways of developing and regenerating hair cells. A) Vestibular hair cells specified early during embryonic period acquire stereocilia, differentiate into subtypes of hair cells, and subsequent innervation. Postnatally generated/regenerated hair cells develop somatic features including differentiation into hair cell subtypes and innervation but were delayed in apical bundle development. B) In the undamaged utricle, majority of Plp1^+^ supporting cells generate type II hair cells, with type I hair cells are only occasionally added. C) In the damaged utricle, majority of Plp1^+^ supporting cells regenerate type II hair cells, type I hair cells are occasionally regenerated. D) By contrast, in the damaged utricle, Lgr5^+^ supporting cells regenerate a similar ratio of type II and type I hair cells. A subset of type I HC^R^ was innervated with Tuj1^+^ calyx (24.4%).

The maturation process of hair cells derived from the prenatal period is orderly with the maturation of bundle and somatic properties seemingly coupled (Fig 12A). Our current and previous data [9] indicate that hair cells generated in vivo in the postnatal utricle have dramatically underdeveloped hair bundles and MET channel function, though seemingly mature for defined properties in the somatic compartment (presence of gDR, gKIR, gH, gKL, lack of gNA, large cell size, negative resting membrane potentials) (Fig 12B–12D). These somatic properties suggest that postnatally re/generated hair cells are comparable to those present in the postnatal utricle, although the underdeveloped stereociliary bundles and MET function imply components of the hair bundle lagged behind in maturation. This interpretation is further supported by the findings that postnatally re/generated hair cells have comparable somatic properties to untraced hair cells (presumably older and derived from the embryonic period). It is therefore possible that overlapping but distinct mechanisms direct the maturation of hair cell apical and somatic domains. In support of this notion, Oshima and colleagues reported that vestibular hair cell-like cells differentiated in vitro from mouse embryonic stem (ES) and iPS cells acquired mechanosensitivity and stereocilia with certain mature features (e.g. staircase pattern), but relatively immature basolateral ion channel properties [20]. Moreover, ectopic cochlear hair cells formed as a result of in utero gene transfer of *Atoh1* to the developing inner ear were crowned with relatively immature stereociliary bundles while displaying age-appropriate conductances and synaptic components on their basolateral surfaces [48]. At present, there is a paucity of knowledge on mechanisms regulating the maturation of individual components of postnatal vestibular hair cells.

### Classification of vestibular hair cells subtypes

Vestibular hair cell subtypes are traditionally characterized according to morphology, molecular markers, electrophysiological properties, and innervation patterns [59,26,16,29]. Recent studies have further characterized the unique basolateral processes of type II hair cells [60,26]. Using markers of type I and II hair cells identified via single cell RNA sequencing [23], we found type I and II hair cells with various morphologies: around 69–79% of the Annexin A4^+^ type II hair cells displayed typical basolateral process, while others were devoid of this feature (S2F Fig). Conversely, type I hair cells showed more uniform morphologies, with the majority (75–100%) being amphora-shaped with long apical necks (Fig 12B). We found occasional Osteopontin^+^ type I hair cells with shorter cell bodies and necks, with nuclei located in the upper third of the epithelium closer to the type II hair cell nuclei level (S2G Fig). Interestingly, we also observed occasional type II hair cells with thick apical necks with nuclei located in the middle third of the epithelium as seen in type I hair cells (S2F Fig). While one may perceive similarities in morphology between some type I and II hair cells, whether their lineages are related remains unclear. Such a relationship between type I and type II hair cells has been previously proposed [61]. In the undamaged tissues, one likely explanation for the various morphologies is that the P30 utricle, though functionally mature, contains a heterogeneous population of hair cells at various developmental stages. Similarly, in the neonatal utricle where new hair cells continue to be generated, hair cells with various morphologies were observed [16]. Cell shapes were further modified as a result of damage, where a noticeable flattening of the sensory epithelium leads to shortening/rounding of both type I and II hair cells (Fig 12C).

Despite the heterogeneous morphologies of Annexin A4^+^ type II hair cells, they displayed remarkably similar channel (gDR and gH) and presynaptic properties, suggesting acquisition of these features may occur before cell morphology becomes more uniform. On the other hand, none of the Osteopontin^+^ type I hair cells derived from Plp1^+^ supporting cells displayed gKL or calyx-type nerve terminals, and only a subset of those from Lgr5^+^ supporting cells showed gKL (28.6%) or calyx nerve terminals (24.4%). Using embryonic and neonatal hair cell development as a reference [14,16], we hypothesize that type I hair cells first express Osteopontin and become amphora-shaped before acquiring gKL, with calyx formation being one of the last maturation steps. In other words, Plp1-traced Osteopontin^+^ type I hair cells, unlike the Lgr5-traced Osteopontin^+^ type I hair cells, failed to display gKL or calyx nerve terminals, and as such are likely to be more immature. Whether these immature type I hair cells further mature over time should be of interest for future studies.

As a cardinal feature of mature hair cells, stereociliary bundles elongate as hair cells mature and gain mechanosensitivity [13,41]. Hair cell subtypes also display different bundle morphologies, with type I hair cells having thicker and longer stereociliary bundles than type II hair cells [62]. Traditionally, bundle length and morphology have been employed as criteria for hair cell identity and maturation. Since results from the current and other studies suggest that hair bundles of postnatally produced hair cells might develop at a different pace compared to other hair cell properties, we propose that bundle features should not be the sole measure of hair cell maturity.

### Supporting cells with distinct competence to regenerate hair cells

In the adult mouse utricle, the ratios of type I and type II hair cells are estimated to be 1.47 and 1.19 in the striola and extrastriola, respectively [46]. While both hair cell types are presumably important for organ function, only regeneration of type II hair cells has been reported in the mature utricle [3–6]. However, our previous and current study suggest that the striolar region in the neonatal utricle uniquely harbors hair cell progenitors capable of regenerating bona fide type I hair cells. The overwhelming majority of Plp1-traced supporting cells occupied the extrastriolar region with few striolar supporting cells also traced, but ~90% traced hair cells displayed type II characteristics and the ~10% Osteopontin^+^ type I hair cells failed to display gKL or calyx innervation. By contrast, Lgr5 becomes highly expressed in striolar supporting cells after damage [9], giving rise to type I hair cells fulfilling morphologic, molecular, electrophysiological, and innervation criteria. Therefore Lgr5^+^ striolar supporting cells may represent a specialized supporting cell population capable of regenerating both type I and II hair cells via mitotic regeneration [9]. In support of the concept that distinct populations of hair cell precursors/progenitors exist, striolar supporting cells are more competent to divide and form ectopic hair cells upon inhibition of Notch signaling [63,42,64].

### Time course of functional recovery and hair cell maturation

Hair cell regeneration is robust in avian auditory and vestibular organs, leading to near-normal densities of hair cells and functional recovery [1]. In the mammalian utricle where regeneration is limited, previous studies examining whether a recovery of function occurs have yielded mixed results [65,66]. In the current study, after hair cell ablation in the neonatal utricle, only 8% of hair cells remained at P15 and 94% of mice lost VsEP responses. At P30 and P60, 36% and 59% of these mice partially regained VsEP thresholds, which coincided and correlated with increases in hair cell numbers (R^2^ = 0.62, Fig 5, S4 Fig). As most of the regenerated hair cells we characterized displayed immature apical stereociliary bundles and poor GTTR uptake, they are unlikely to be fully functional or to significantly contribute to the observed functional recovery. Since many hair cells derived from the Plp1-traced and Lgr5-traced supporting cells appear nonfunctional, it is possible that other supporting cells/progenitor cells contribute to functional hair cells that are produced in the postnatal utricle.

Overall, we estimate that postnatally generated hair cells with immature stereociliary bundles constitute ~13% of the hair cell population. It is possible that these hair cells act as a reserve population that replenishes lost hair cells in response to damage. Alternatively, they may function to replace hair cells lost during the homeostatic turnover of hair cells in the adult utricle [60]. Moreover, surviving hair cells and a change in the innervating patterns may permit the activation of neurons from fewer stimulated hair cells, which in turn contribute to functional recovery. The *Pou4f3-DTR* mouse is an ideal model to further investigate these possibilities.

In summary, both hair cells generated in the postnatal utricle and hair cells regenerated after damage demonstrated features of mature somatic properties yet immature apical stereociliary bundles and MET function. Based on these results, we propose a new classification of postnatal vestibular hair cells independent of apical bundle morphology, which warrants further characterization in the normal and damaged postnatal utricle. Moreover, our data may guide future efforts in inner ear regeneration, including delineating the relationship between hair cell regeneration and vestibular function.

## Methods

### Ethics statement

All protocols (#18606) were approved by the Animal Care and Use Committee of the Stanford University School of Medicine.

### Mice

*Plp1-CreERT* (Jackson Laboratory, #5975)[67], *Lgr5-EGFP-CreERT2* (Jackson Laboratory, #8875)[68], *Rosa26R-tdTomato* (Jackson Laboratory, #7908)[69], and *Pou4f3-DTR* [44] mice were used. For Cre activation, tamoxifen (0.075 mg/g, dissolved in corn oil; Sigma) was given via IP injection to neonatal mice. Diphtheria toxin (4 ng/g intramuscular [IM], EMD Millipore) was used for hair cell ablation.

### Immunohistochemistry

Utricles were fixed for 40 minutes in 4% paraformaldehyde (in PBS, pH 7.4; Electron Microscopy Services) at room temperature. Tissues were blocked with 5% donkey serum, 0.1% TritonX-100, 1% bovine serum albumin (BSA), and 0.02% sodium azide (NaN_3_) in PBS at pH 7.4 for 1–2 hours at room temperature, followed by incubation with primary antibodies diluted in the same blocking solution overnight at 4°C. The next day, after washing with PBS, tissues were incubated with secondary antibodies diluted in 0.1% TritonX-100, 0.1% BSA, and 0.02% NaN_3_ solution in PBS for 2 hours at room temperature. After PBS washing, tissues were mounted in antifade Fluorescence Mounting Medium (DAKO) and coverslipped. Antibodies against the following markers were used: Myosin7a (1:1000; Proteus Bioscience or Labome), Sox2 (1:400, Santa Cruz Biotechnology), Sox2 (1:250; or Alexa Fluor 488-conjugated, 1:20; R&D), Tuj1 (1:1000; Neuromics), Ctbp2 (1:1000; BD Transduction Laboratories), Annexin A4 (1:200; R&D), Osteopontin (1:200; R&D) and Mapt (1:200, Cell Signaling). Secondary antibodies were conjugated with FITC (1:500, Invitrogen), TRITC (1:500, Invitrogen), CY5 (1:250, Invitrogen) or Alexa 405 (1:250, Abcam). Fluorescence-conjugated phalloidin (1:1000; Sigma) and DAPI (1:10000; Invitrogen) were also used. Utricles were incubated with Texas Red-conjugated gentamicin (GTTR) for 1 hour with 1:100 of stock solution (0.85 mg/mL) [70,71]. Images were acquired using epifluorescent or confocal microscopy (LSM700 or LSM880, Zeiss) and analyzed with ImageJ (64 bit), Fiji (NIH) [72], and Photoshop CS6 (Adobe Systems). Optical slices 1 μm thick were used in z-stack images.

### Cellular quantification and statistics

Cells were quantified from z-stack images of 10,000 μm^2^ or the whole sensory epithelium using ImageJ (64 bit) unless otherwise stated. Images were taken from 1–2 representative areas from striolar and extrastriolar regions or the whole sensory epithelium for analysis. For all experiments, n values represent the number of cells or mice examined unless otherwise stated. Statistical analyses were conducted using Microsoft Excel (Microsoft), GraphPad Prism 7.0 software (GraphPad), Origin (Microcal) and scripts written in MATLAB when necessary. Two-tailed, unpaired Student *t* tests and one-way ANOVA by Tukey’s and Kruskal Wallis-Dunn's multiple comparison tests were used to determine statistical significance. *p* < 0.05 was considered statistically significant. Data shown as mean ± SD.

### Genotyping

PCR was performed to genotype transgenic mice using genomic DNA. DNA was isolated by adding 200 μl of 50 mM NaOH to cut tail tips, incubating at 98°C for 1 hour, and then adding 20 μl of 1 M Tris-HCl. Primers used are listed in S1 Table.

### Vestibular physiology

VsEPs were recorded from mice at various ages (P15, P30 and P60) as previously described [45]. Briefly, mice were first anesthetized with a 1:1 cocktail of ketamine (60 to 100 mg/kg) and xylazine (6 to 10 mg/kg). Subcutaneous stainless steel electrodes were then placed, and a head clip was used to secure the head to the mechanical shaker used to deliver linear motion stimuli along the naso-occipital axis. Vertical motion of the shaker was monitored with an accelerometer (model no. 1018, Vibra-Metrics) and adjusted to produce the stimulus waveforms. Jerk stimuli ranging from 0.1 to 2.0 g/ms were presented (g/ms where 1 g = 9.8 m/s^2^) [45]. Signals were amplified and filtered. Responses from normal and inverted stimulus polarities were collected and added together for a total of 256 sweeps for each waveform. A masker (90 dB SPL; bandwidth 50 Hz to 50 kHz) from a free-field speaker driver (model no. FF1, Tucker Davis Technologies) was used to prevent responses from the auditory components of cranial nerve VIII. All primary response parameters were blindly quantified via three components: threshold (g/ms), P1 latency (ms) and P1-N1 amplitude (μV). Thresholds were defined as the stimulus intensity halfway between the minimum response intensity and the maximum intensity that failed to elicit a response. Latencies were measured as the time to onset of the stimulus for the first positive response peak (P1). Amplitudes represented peak-to-peak magnitudes between P1 and N1 waveforms. Whereas the first positive and negative response peaks (P1 and N1, respectively) reflect activity of the peripheral vestibular nerve, peaks beyond N1 reflect activity of the brainstem and central vestibular relays [45].

### Electrophysiology

Utricular tissues were dissected from mice at ages denoted and secured in a recording chamber where epifluorescence, cell morphology and location could be observed for identification and classification. An Olympus, BX50 microscope with a 100x (1.0 NA) objective was used for all observations. Tissues were isolated and incubated for recording in a solution containing 140 mM NaCl, 5.4 mM KCl, 2.8 mM CaCl_2_, 1 mM MgCl_2_, 10 mM 4-(2-hydroxyethyl)-1-piperazineethanesulfonic acid (HEPES), 6 mM D-glucose, 2 mM creatine, 2 mM ascorbate, 2 mM Na pyruvate, pH 7.4 adjusted with NaOH. 100 nM apamin was included to block the SK K^+^ channel. The patch pipette contained 135 mM KCl, 1 mM ethylene glycol-bis (B-aminoethyl ether)-N,N,N’,N’-tetraacetic acid (EGTA), 3 mM MgCl_2_, 3 mM Na_2_ Adenosine triphosphate (ATP), 5 mM creatine phosphate, 10 mM HEPES, and 5 mM ascorbate. For isolation of the calcium current and for capacitance measurements, KCl was replaced with 90 mM CsCl and 15 mM tetraethylammonium (TEA). Additionally, 3 mM CsCl was added to the external solution to block inward rectifier currents.

Whole-cell patch-clamp recordings were elicited using standard technology including axopatch 200b amplifier, an A/D, D/A board (IOTech) driven by JClamp software (SciSoft). The various conductances were grossly separated using specific voltage protocols as outlined below.

For characterizing the delayed rectifier currents IDR, cells were voltage clamped at -69 mV and stepped in 10 mV increments for 400 ms between -94 mV and 24 mV. Activation curves were generated from the tail current responses using peak current at 3 ms following the end of the step. To convert from current to conductance we assayed reversal potential using a protocol that held the cell at -89 mV for 10 ms, stepped to 14mV for 250 ms and then stepped between -116 mV and 14 mV in 5 mV increments for 50 ms after which it returned to -89 mV. A plot of peak current against voltage during the 5 mV incremental steps allows for estimation of the reversal potential. From this protocol, the reverse potential is estimated at -73 mV.

We used IKL to define type I hair cells. The absence of IKL and the presence of IH were used to define non-type I, and thus a type II, hair cell phenotype. We did not assess IH in the presence of IKL.

Characterization of IKL involved voltage-clamping cells at -89 mV, stepping to -124 mV for 250 ms and then stepping between -124 mV and -45 mV in 5 mV increments for 150 ms. A trace showing the classical deactivation of the IKL is shown in S7F Fig. Plots of peak current versus voltage step were generated and converted to conductance plots after the reversal potential was identified from a protocol where a cell was voltage-clamped at -89 mV depolarized to -69 mV for 250 ms and then stepped between -124 and -41 mV in 5 mV for 150 ms, returning then to -89 mV. From this protocol, the reverse potential is estimated at -73 mV.

For characterizing the inward rectifier current IH, cells were voltage clamped at -69 mV, hyperpolarized between -74 and -156 mV for 400 ms, and then back to -69 mV. Current-voltage plots were generated for maximal current elicited during hyperpolarization. These were converted to conductance plots by obtaining reversal potentials using a protocol that voltage-clamped a cell at -104 mV for 500 ms and then stepped between -120 and -55 mV for 5 ms, at which time it returned to -84 mV. Current-voltage plots identified the reversal potential that allowed for conductance estimates as shown in the text. From this protocol, the reverse potential is estimated at -62 mV.

From conductance-voltage plots of IDR, we found values for maximal conductance, half-activation voltage and slope of the Boltzmann function using methods detailed in S7 Fig. Similar methods were used for IH and IKL.

Calcium currents were isolated by replacing potassium with a cesium-based intracellular solution using a protocol that voltage-clamped the hair cell at -69 mV and stepped for 10 ms between -69 mV and 26 mV in 10 mV increments. Current-voltage plots were generated from peak current responses and maximal current and half activating voltage were extracted from these plots.

To assess functional activity of presynaptic sites, vesicle fusion was monitored using a dual sine wave technique as previously described [39,40]. Hair cells were depolarized to the voltage of maximal calcium current for 3 seconds, and changes in capacitance were monitored in real time. The sine wave amplitude was 30 mV and the frequencies selected were 6250 and 3125 Hz. Protocols were run after 10 minutes to ensure equilibration of internal solution and stabilization of calcium currents.

## Supporting information

S1 Fig Characteristics of postnatally generated hair cells in the mouse utricle. A) Whole mount utricle from P30 *Plp1*^*CreERT/+*^*; Rosa26R*^*tdTomato/+*^ mice treated with tamoxifen at P3 (early tracing) to fate-map supporting cells. Most supporting cells were labeled with tdTomato in both the lateral and medial extrastriolar regions (LES and MES). Some supporting cells in the striolar (S) region (dashed line) were also traced. Boxes of solid line and dashed line represent typical locations where high magnification pictures of the extrastriola and striola were captured. B) No tdTomato^+^/Myosin7a^+^ hair cells were detected in the striola 2 days after early tracing. C) At P30, many traced hair cells (asterisks) were found in the striola. D) When tracing was initiated at P8 (late tracing), a few traced hair cells (asterisks) were noted in the striola at P30. E) Percentage of traced hair cells increased significantly at P30 compared to P5 after early tracing. There were fewer traced hair cells when tracing began at P8. F) *Plp1*^*CreERT/+*^*; Rosa26R*^*tdTomato/+*^ mice were treated with corn oil at P3. No hair cells and rare supporting cells were tdTomato-labeled at P30 (*n =* 665 and 608 hair cells, 954 and 946 supporting cells from 3 mice in the extrastriola and striola). Data shown as mean ± SD and compared using Student *t* tests. **p < 0.01, *p < 0.05. *n* = 663–2,562 hair cells from 4–16 mice. Scale bars: A) 100 μm; B-D, F) 20 μm. The underlying data can be found within S1 Data.
(TIF)

S2 Fig Early and late postnatally generated hair cells acquire characteristics of type II and I hair cells. A) *Plp1*^*CreERT/+*^*; Rosa26R*^*tdTomato/+*^ mice were treated with tamoxifen at P3 (early tracing) and P8 (late tracing) to fate-map supporting cells. B-C) Most labeled hair cells in the striola from early and late tracing expressed the type II hair cell marker ANXA4 (asterisks). D-E) Traced hair cells in the striola from early and late tracing were occasionally labeled with type I hair cell marker OPN (arrowheads). Shown are orthogonal views of representative cells in B-E). F-G) Representative examples of various morphologic subtypes of type II and type I hair cells which were generated postnatally. H) Representative orthogonal views of type I hair cells (arrowhead and dashed lines) with OPN and Tuj1 staining. I) Cartoon depicting Mapt^+^ (green) type II hair cells. J-K) Representative images of Mapt^+^/ANXA4^+^/tdTomato^+^/Myosin7a^+^ (arrowhead) hair cells in the extrastriola and striola (from late tracing experiments). Scale bar: B-E, J-K) 20 μm. ANXA4, Annexin A4; OPN, Osteopontin.
(TIF)

S3 Fig Synaptic properties of postnatally generated hair cells. A-B) Representative images of tdTomato^+^/Myosin7a^+^ hair cells (asterisks and dashed lines) from early and later tracing with associated Tuj1^+^ neurites (arrowheads in orthogonal views, *n =* 18 cells from 3 mice and 18 cells from 9 mice for early and late tracing). C-D) Expression of Ctbp2 on the basolateral surfaces of traced hair cells (arrowhead). Shown are XY and orthogonal views of cells of interests in boxes, *n =* 19 cells from 3 mice and 25 cells from 6 mice for early and late tracing. E) Quantification of Ctbp2^+^ puncta in tdTomato^+^/Myosin7a^+^ hair cells in the striola. No significantly difference was found between early and late tracing. F-H) Three groups of hair cells (HC^PG3^, HC^PG8^ and untraced hair cells, *n =* 17, 8, and 4 cells, respectively) were depolarized to the voltage of maximal calcium current for 3 seconds and changes in capacitance monitored in real time. I-J) 75–100% of the recorded cells had greater than 50 fF of capacitance change. No differences in maximal release were observed among groups. Data shown as mean ± SD, compared using Student *t* tests and one-way ANOVA by Kruskal Wallis-Dunn's multiple comparison tests. Scale bars: A-D) 20 μm. The underlying data can be found within S1 Data.
(TIF)

S4 Fig Hair cell number and vestibular function recover after hair cell ablation. A-E) High magnification images showing loss of striolar hair cells at P15 after DT treatment at P1, followed by a partial recovery at P30 and P60. F-G) Normalized (to age-matched, undamaged controls) percentage of Myosin7a^+^ hair cell density in the extrastriola and striola (*n =* 6 at P15, 15 at P30 and 11 at P60). H) Normalized (to age-matched, undamaged controls) percentage of Myosin7a^+^ sensory epithelium area at P15, P30 and P60. I) In comparison to P15, P1 latency values significantly decreased at P30 and P60, but still significantly higher than age-matched controls. J) Relative to P15, P1-N1 amplitudes remained lower than normal at P30 and P60 (*n =* 48 at P15, 39 at P30 and 27 at P60). K) Correlation between hair cell number and VsEP thresholds from P15, P30 and P60 mice (*n =* 4 at P15, 4 at P30 and 14 at P60). Data shown as mean ± SD, compared using Student *t* tests and one-way ANOVA by Tukey’s multiple comparison test. ***p < 0.001, **p < 0.01, *p < 0.05. Scale bars: A-E) 20 μm. The underlying data can be found within S1 Data.
(TIF)

S5 Fig Characteristics of Plp1-traced regenerated hair cells. A) Whole mount utricles from *Plp1*^*CreERT/+*^*; Rosa26R*^*tdTomato/+*^ mice treated with DT at P1, followed by tamoxifen at P8 to fate-map Plp1^+^ cells. Organs were examined at P30. B-C) Representative images showing traced supporting cells in the extrastriolar and regions in the P30 damaged utricle. D-E) Representative images of different morphological subtypes of type II and type I hair cells regenerated from Plp1^+^ supporting cells. F-G) Representative confocal images of Mapt^+^/ANXA4^+^/tdTomato^+^/Myosin7a^+^ (arrowhead) and Mapt^-^/ANXA4^+^/tdTomato^+^/Myosin7a^+^hair cells (arrow) in the extrastriola and striola of damaged utricles. H-J) Representative tracings showing changes in capacitance in HC^R^, HC^PG8^ and untraced hair cells (*n =* 3–14 cells), which were depolarized to the voltage of maximal calcium current for 3 seconds. K-L) More than 85% HC^R^, HC^PG8^ and untraced hair cells showed greater than 50 fF of capacitance change. No significant differences in maximal release were observed among groups. Data shown as mean ± SD, compared using one-way ANOVA by Kruskal Wallis-Dunn's multiple comparison tests. Scale bars: A) 10 μm. B-C, F-G) 20 μm. The underlying data can be found within S1 Data.
(TIF)

S6 Fig Characteristics of regenerated hair cells from Lgr5+ supporting cells. A) Schematic of the genetic approach to ablate hair cells and fate-map Lgr5^+^ cells in vivo. B-D) In undamaged control tissues, few traced hair cells expressed type II hair cell marker ANXA4 (arrow) and no OPN^+^ traced hair cells with Tuj1^+^ calyx were found (*n =* 10). Arrowheads highlight Tuj1^+^ neural elements. D) Lgr5-traced hair cells (arrow) immunonegative for OPN and Tuj1^+^ neurites. Inset shows orthogonal view of traced hair cells with associated innervation (arrowheads). E) Representative images of different morphologic subtypes of regenerated type I hair cells from Lgr5^+^ supporting cells. F-H) Represent calcium currents from Lgr5-traced HC^R^ (black) and untraced hair cells from damaged (red) and undamaged (blue) utricles. I-J) Peak current responses and maximal current and half activating voltage were not statistically different among groups (*n =* 5–8 cells). K-M) Representative tracings of real-time changes in capacitance in Lgr5-traced HC^R^ (black), untraced hair cells from damaged (red) and undamaged (blue) when they were depolarized to the voltage of maximal calcium current for 3 seconds. N) About 50% of the Lgr5-traced HC^R^ and >85% of untraced hair cells had greater than 50 fF of capacitance change (*n =* 5–8 cells). No differences in maximal release were observed among groups. Data shown as mean ± SD, compared using one-way ANOVA by Kruskal Wallis-Dunn's multiple comparison tests. Scale bars: B-D) 20 μm. The underlying data can be found within S1 Data. HC^R^, regenerated hair cell; OPN, Osteopontin.
(TIF)

S7 Fig Methods used for identifying the conductance plot from which half-activation and steepness plots were generated. A) Voltage clamp data of standard protocol for eliciting IDR, protocol timing shown above the current responses. B) Currents at the time point indicated by line in (A) against stimulus voltage. C) Currents from a protocol used to identify the reversal potential for the primary conductance, stimulus timing shown above currents. The cell was stepped to -4mV, potential eliciting a large outward current (A, B) and then repolarized to potentials between -124 and 30 mV (Vm). D) The reversal potential obtained in D (-78 mV), from plotting the data in (C) was used in E to generate a conductance plot where g = I/(Vm-Vr). E) Shown is an example of a single IKL response. The underlying data can be found within S1 Data.
(TIF)

S1 Table Primers for genotyping. (XLSX)

S2 Table Electrophysiological properties of postnatally generated and regenerated hair cells. (XLSX)

S3 Table Percent of hair cells with markers of type I and II hair cells. (XLSX)

S4 Table Quantification of Mapt- and Annexin A4-positive cells in undamaged and damaged P30 utricles. (XLSX)

S1 Data Original data presented in main and supplemental figures. (XLSX)

## Abbreviations

ANXA4: Annexin A4
DT: diphtheria toxin
DTR: diphtheria toxin receptor
E: embryonic day
ES: embryonic stem
G: conductance
gDR: delayed rectifier potassium conductance
gH: slow inward rectifier non-specific cation conductance
gKL: low-voltage-activated, delayed rectifier potassium conductance
gNA: voltage-gated sodium conductance
gKIR: inward rectifier potassium conductance
gK1: fast inward rectifier potassium conductance
GTTR: Texas-Red conjugated gentamicin
HC^PG^: postnatally generated hair cell
HC^R^: regenerated hair cell
I: current
IM: intramuscular
IP: intraperitoneal
iPS: induced pluripotent stem
MET: mechanotransduction
OPN: Osteopontin
P: postnatal day
VsEP: vestibular-evoked potential
