# Supplementary figures and images for: Uncoordinated maturation of developing and regenerating postnatal mammalian vestibular hair cells
Source: PLoS Biol. 2019 Jul 1;17(7):e3000326. doi: 10.1371/journal.pbio.3000326 (PMC6602158; doi:10.1371/journal.pbio.3000326)

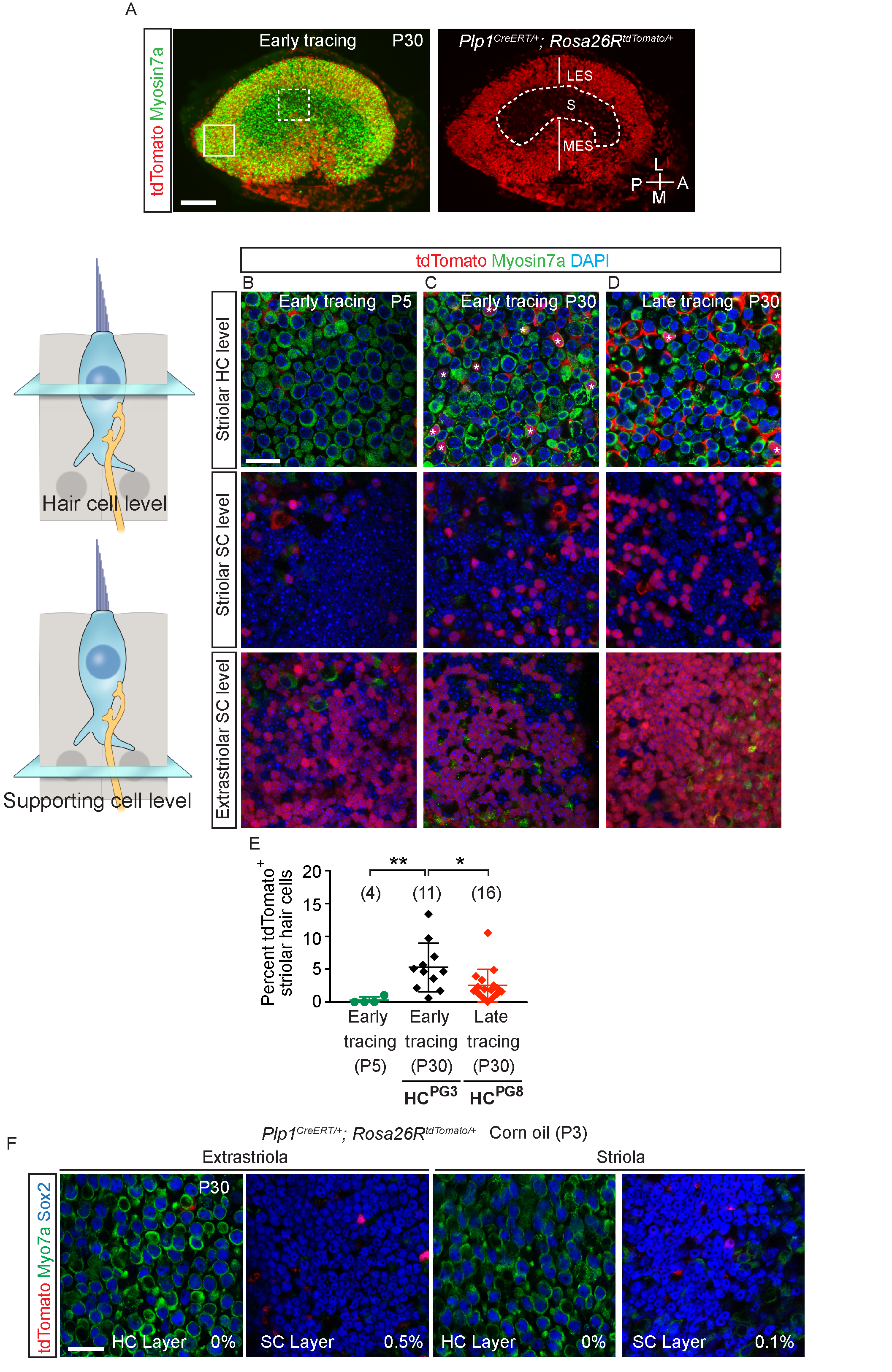

Supplement: S1 Fig — A) Whole mount utricle from P30 Plp1CreERT/+; Rosa26RtdTomato/+ mice treated with tamoxifen at P3 (early tracing) to fate-map supporting cells. Most supporting cells were labeled with tdTomato in both the lateral and medial extrastriolar regions (LES and MES). Some supporting cells in the striolar (S) region (dashed line) were also traced. Boxes of solid line and dashed line represent typical locations where high magnification pictures of the extrastriola and striola were captured. B) No tdTomato+/Myosin7a+ hair cells were detected in the striola 2 days after early tracing. C) At P30, many traced hair cells (asterisks) were found in the striola. D) When tracing was initiated at P8 (late tracing), a few traced hair cells (asterisks) were noted in the striola at P30. E) Percentage of traced hair cells increased significantly at P30 compared to P5 after early tracing. There were fewer traced hair cells when tracing began at P8. F) Plp1CreERT/+; Rosa26RtdTomato/+ mice were treated with corn oil at P3. No hair cells and rare supporting cells were tdTomato-labeled at P30 (n = 665 and 608 hair cells, 954 and 946 supporting cells from 3 mice in the extrastriola and striola). Data shown as mean ± SD and compared using Student t tests. **p < 0.01, *p < 0.05. n = 663–2,562 hair cells from 4–16 mice. Scale bars: A) 100 μm; B-D, F) 20 μm. The underlying data can be found within S1 Data. (TIF) [file pbio.3000326.s001.tif]

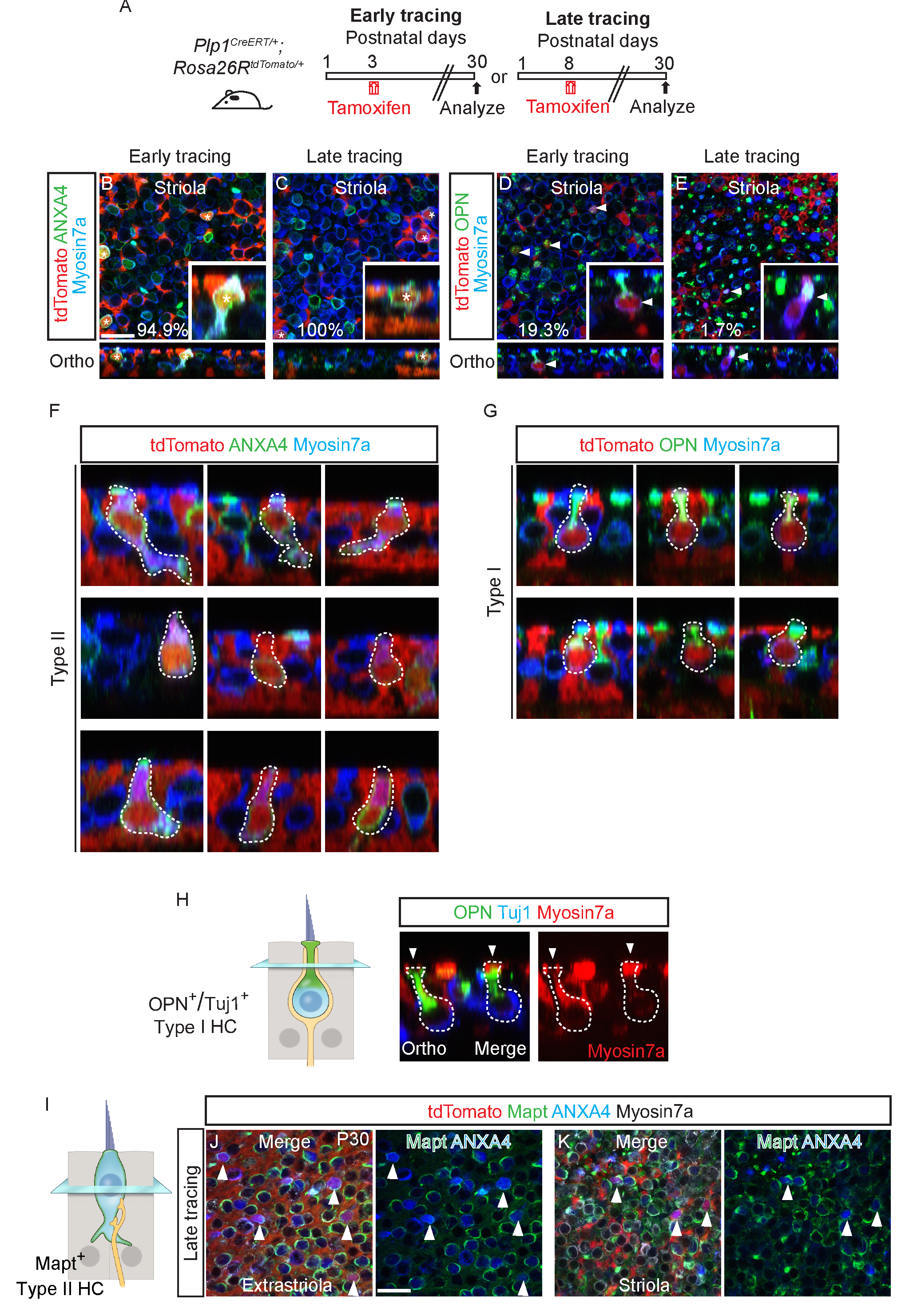

Supplement: S2 Fig — A) Plp1CreERT/+; Rosa26RtdTomato/+ mice were treated with tamoxifen at P3 (early tracing) and P8 (late tracing) to fate-map supporting cells. B-C) Most labeled hair cells in the striola from early and late tracing expressed the type II hair cell marker ANXA4 (asterisks). D-E) Traced hair cells in the striola from early and late tracing were occasionally labeled with type I hair cell marker OPN (arrowheads). Shown are orthogonal views of representative cells in B-E). F-G) Representative examples of various morphologic subtypes of type II and type I hair cells which were generated postnatally. H) Representative orthogonal views of type I hair cells (arrowhead and dashed lines) with OPN and Tuj1 staining. I) Cartoon depicting Mapt+ (green) type II hair cells. J-K) Representative images of Mapt+/ANXA4+/tdTomato+/Myosin7a+ (arrowhead) hair cells in the extrastriola and striola (from late tracing experiments). Scale bar: B-E, J-K) 20 μm. ANXA4, Annexin A4; OPN, Osteopontin. (TIF) [file pbio.3000326.s002.tif]

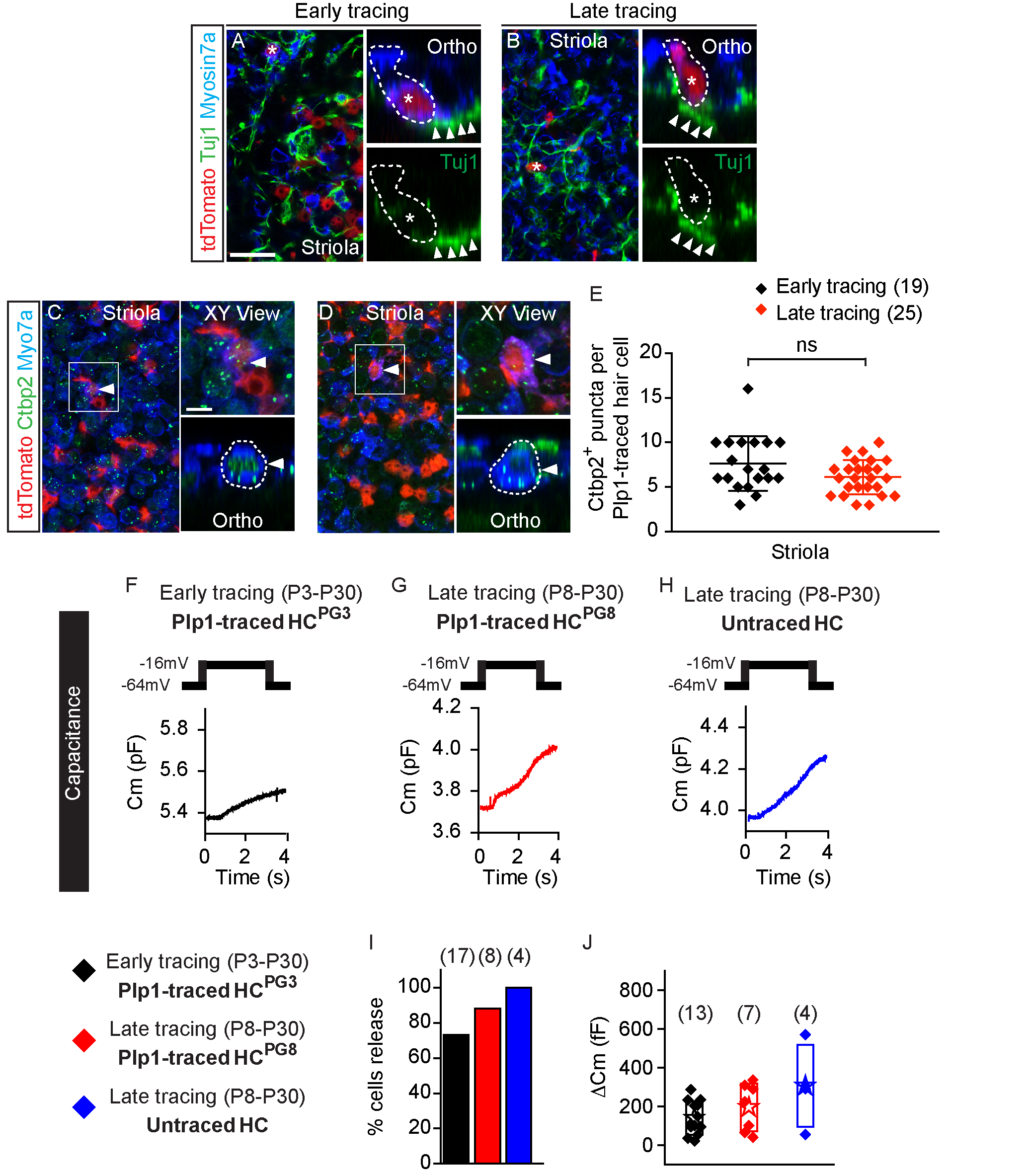

Supplement: S3 Fig — A-B) Representative images of tdTomato+/Myosin7a+ hair cells (asterisks and dashed lines) from early and later tracing with associated Tuj1+ neurites (arrowheads in orthogonal views, n = 18 cells from 3 mice and 18 cells from 9 mice for early and late tracing). C-D) Expression of Ctbp2 on the basolateral surfaces of traced hair cells (arrowhead). Shown are XY and orthogonal views of cells of interests in boxes, n = 19 cells from 3 mice and 25 cells from 6 mice for early and late tracing. E) Quantification of Ctbp2+ puncta in tdTomato+/Myosin7a+ hair cells in the striola. No significantly difference was found between early and late tracing. F-H) Three groups of hair cells (HCPG3, HCPG8 and untraced hair cells, n = 17, 8, and 4 cells, respectively) were depolarized to the voltage of maximal calcium current for 3 seconds and changes in capacitance monitored in real time. I-J) 75–100% of the recorded cells had greater than 50 fF of capacitance change. No differences in maximal release were observed among groups. Data shown as mean ± SD, compared using Student t tests and one-way ANOVA by Kruskal Wallis-Dunn's multiple comparison tests. Scale bars: A-D) 20 μm. The underlying data can be found within S1 Data. (TIF) [file pbio.3000326.s003.tif]

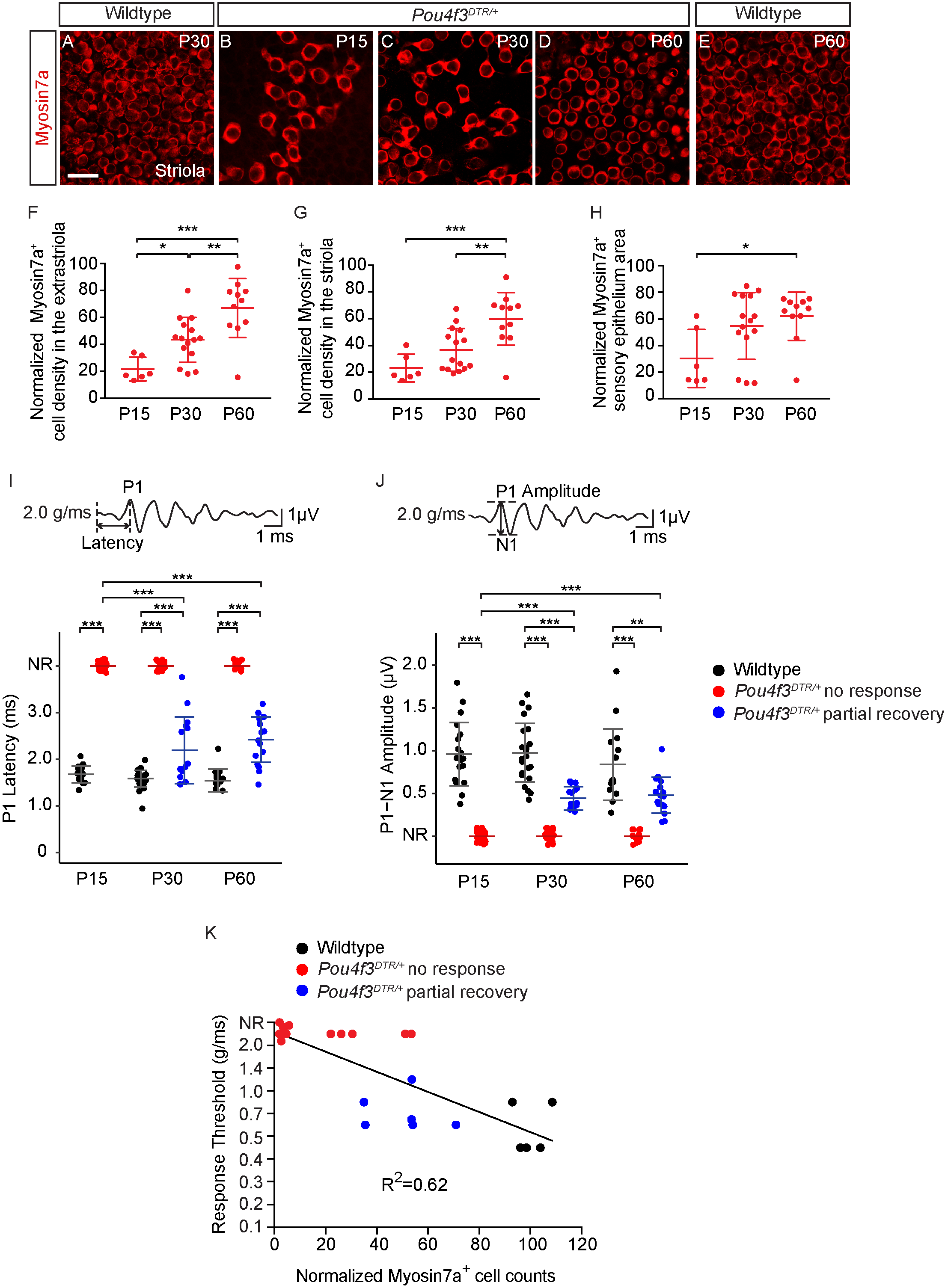

Supplement: S4 Fig — A-E) High magnification images showing loss of striolar hair cells at P15 after DT treatment at P1, followed by a partial recovery at P30 and P60. F-G) Normalized (to age-matched, undamaged controls) percentage of Myosin7a+ hair cell density in the extrastriola and striola (n = 6 at P15, 15 at P30 and 11 at P60). H) Normalized (to age-matched, undamaged controls) percentage of Myosin7a+ sensory epithelium area at P15, P30 and P60. I) In comparison to P15, P1 latency values significantly decreased at P30 and P60, but still significantly higher than age-matched controls. J) Relative to P15, P1-N1 amplitudes remained lower than normal at P30 and P60 (n = 48 at P15, 39 at P30 and 27 at P60). K) Correlation between hair cell number and VsEP thresholds from P15, P30 and P60 mice (n = 4 at P15, 4 at P30 and 14 at P60). Data shown as mean ± SD, compared using Student t tests and one-way ANOVA by Tukey’s multiple comparison test. ***p < 0.001, **p < 0.01, *p < 0.05. Scale bars: A-E) 20 μm. The underlying data can be found within S1 Data. (TIF) [file pbio.3000326.s004.tif]

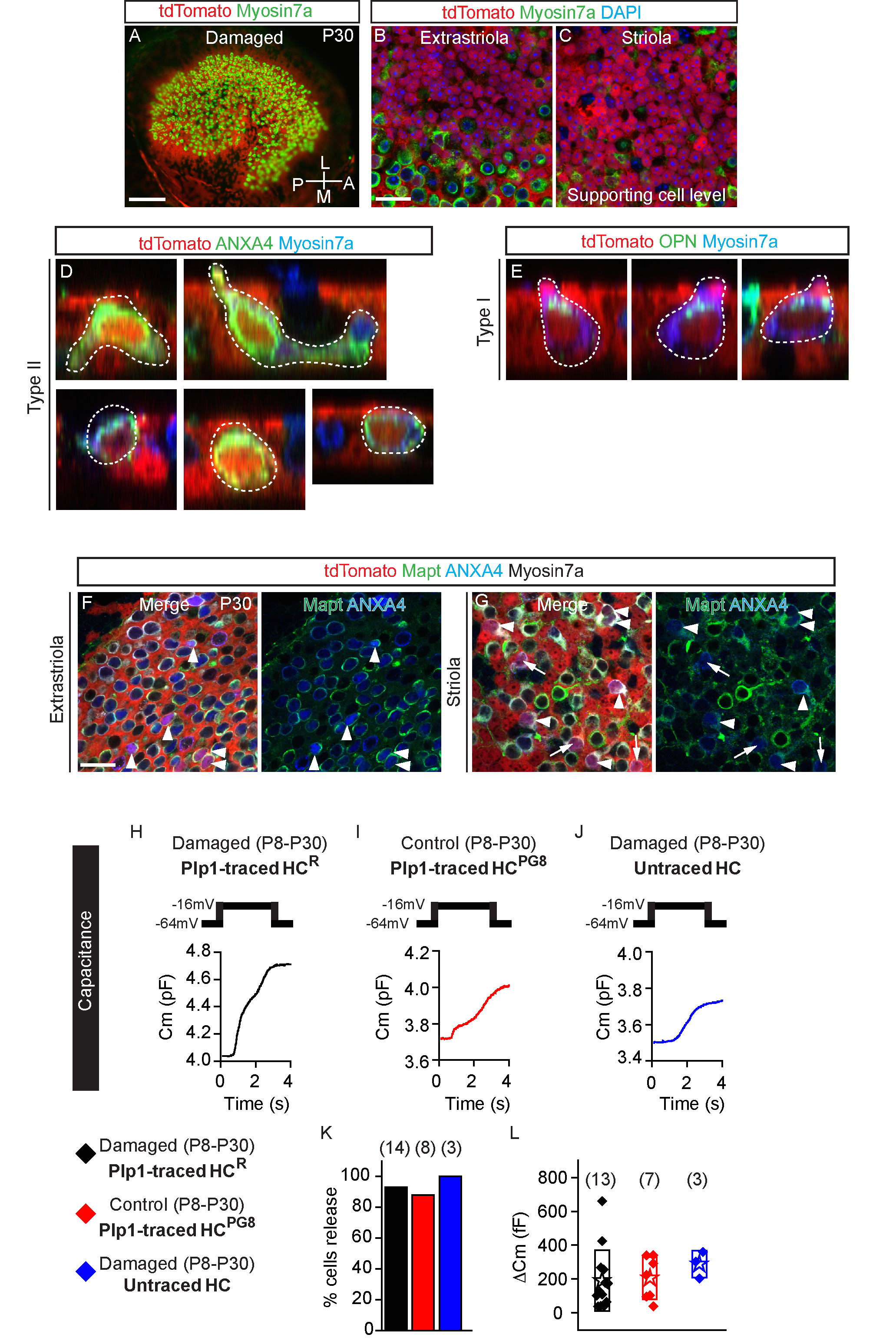

Supplement: S5 Fig — A) Whole mount utricles from Plp1CreERT/+; Rosa26RtdTomato/+ mice treated with DT at P1, followed by tamoxifen at P8 to fate-map Plp1+ cells. Organs were examined at P30. B-C) Representative images showing traced supporting cells in the extrastriolar and regions in the P30 damaged utricle. D-E) Representative images of different morphological subtypes of type II and type I hair cells regenerated from Plp1+ supporting cells. F-G) Representative confocal images of Mapt+/ANXA4+/tdTomato+/Myosin7a+ (arrowhead) and Mapt-/ANXA4+/tdTomato+/Myosin7a+hair cells (arrow) in the extrastriola and striola of damaged utricles. H-J) Representative tracings showing changes in capacitance in HCR, HCPG8 and untraced hair cells (n = 3–14 cells), which were depolarized to the voltage of maximal calcium current for 3 seconds. K-L) More than 85% HCR, HCPG8 and untraced hair cells showed greater than 50 fF of capacitance change. No significant differences in maximal release were observed among groups. Data shown as mean ± SD, compared using one-way ANOVA by Kruskal Wallis-Dunn's multiple comparison tests. Scale bars: A) 10 μm. B-C, F-G) 20 μm. The underlying data can be found within S1 Data. (TIF) [file pbio.3000326.s005.tif]

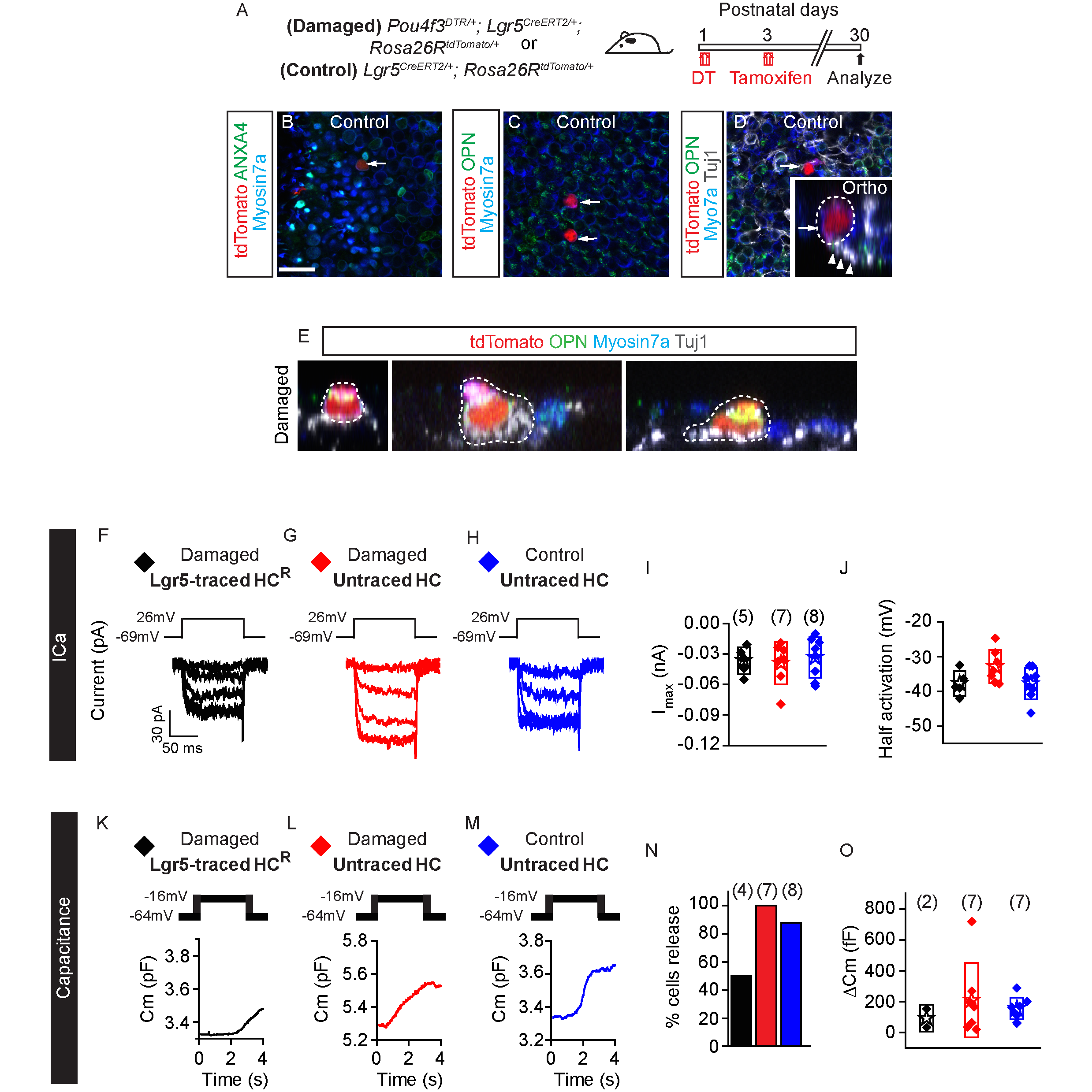

Supplement: S6 Fig — A) Schematic of the genetic approach to ablate hair cells and fate-map Lgr5+ cells in vivo. B-D) In undamaged control tissues, few traced hair cells expressed type II hair cell marker ANXA4 (arrow) and no OPN+ traced hair cells with Tuj1+ calyx were found (n = 10). Arrowheads highlight Tuj1+ neural elements. D) Lgr5-traced hair cells (arrow) immunonegative for OPN and Tuj1+ neurites. Inset shows orthogonal view of traced hair cells with associated innervation (arrowheads). E) Representative images of different morphologic subtypes of regenerated type I hair cells from Lgr5+ supporting cells. F-H) Represent calcium currents from Lgr5-traced HCR (black) and untraced hair cells from damaged (red) and undamaged (blue) utricles. I-J) Peak current responses and maximal current and half activating voltage were not statistically different among groups (n = 5–8 cells). K-M) Representative tracings of real-time changes in capacitance in Lgr5-traced HCR (black), untraced hair cells from damaged (red) and undamaged (blue) when they were depolarized to the voltage of maximal calcium current for 3 seconds. N) About 50% of the Lgr5-traced HCR and >85% of untraced hair cells had greater than 50 fF of capacitance change (n = 5–8 cells). No differences in maximal release were observed among groups. Data shown as mean ± SD, compared using one-way ANOVA by Kruskal Wallis-Dunn's multiple comparison tests. Scale bars: B-D) 20 μm. The underlying data can be found within S1 Data. HCR, regenerated hair cell; OPN, Osteopontin. (TIF) [file pbio.3000326.s006.tif]

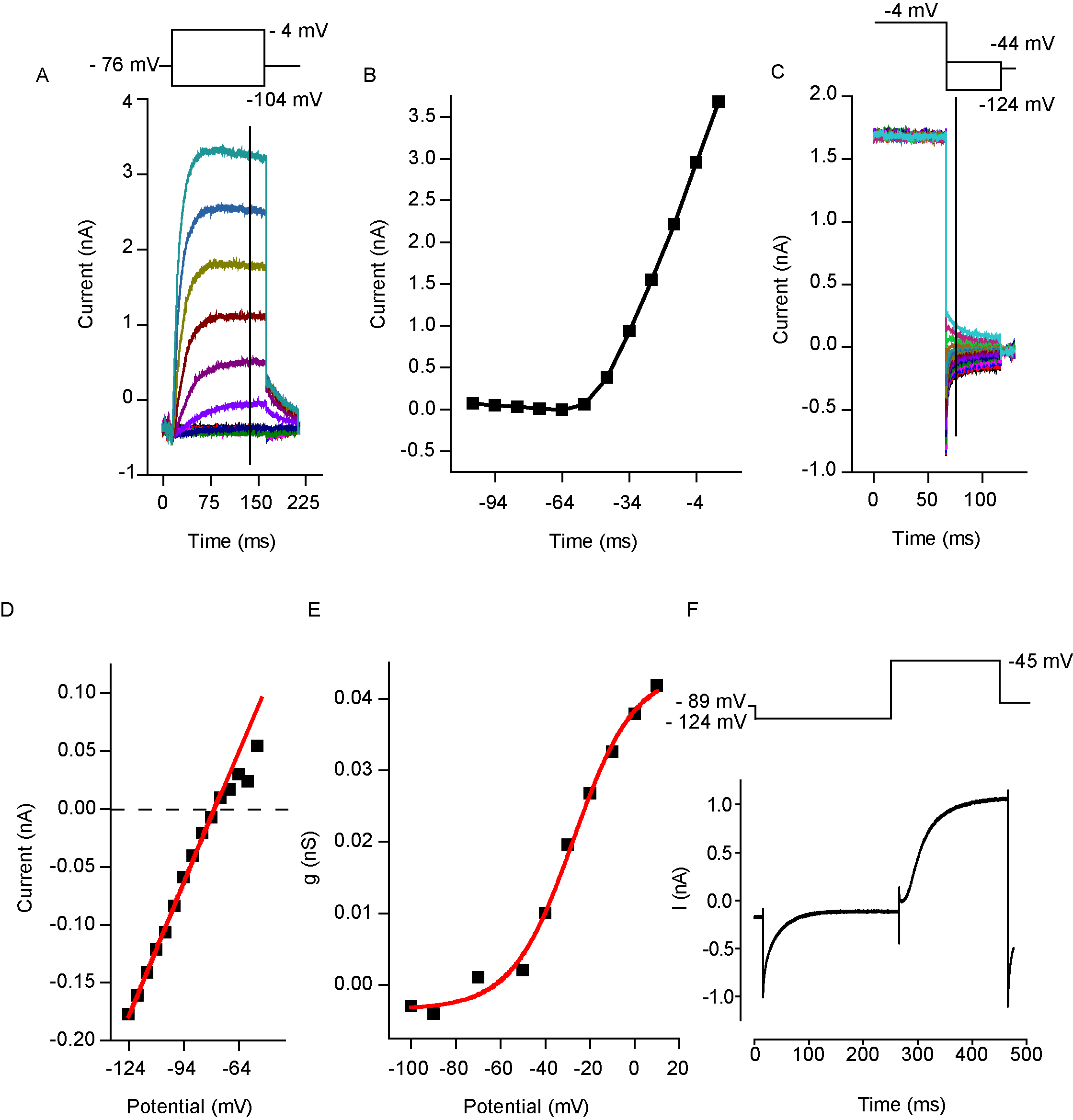

Supplement: S7 Fig — A) Voltage clamp data of standard protocol for eliciting IDR, protocol timing shown above the current responses. B) Currents at the time point indicated by line in (A) against stimulus voltage. C) Currents from a protocol used to identify the reversal potential for the primary conductance, stimulus timing shown above currents. The cell was stepped to -4mV, potential eliciting a large outward current (A, B) and then repolarized to potentials between -124 and 30 mV (Vm). D) The reversal potential obtained in D (-78 mV), from plotting the data in (C) was used in E to generate a conductance plot where g = I/(Vm-Vr). E) Shown is an example of a single IKL response. The underlying data can be found within S1 Data. (TIF) [file pbio.3000326.s007.tif]
